# Mitochondrial quality, dynamics and functional capacity in Parkinson’s disease cybrid cell lines selected for Lewy body expression

**DOI:** 10.1186/1750-1326-8-6

**Published:** 2013-01-26

**Authors:** Emily N Cronin-Furman, M Kathleen Borland, Kristen E Bergquist, James P Bennett, Patricia A Trimmer

**Affiliations:** 1 Neuroscience Graduate Program, University of Virginia, Charlottesville, VA, 22908, USA; 2 Parkinson’s and Movement Disorders Center, Virginia Commonwealth University, Richmond, VA, 23298, USA; 3 Department of Neurology, University of Virginia, Charlottesville, VA, 22908, USA; 4 Department of Neurology, Virginia Commonwealth University, Richmond, VA, 23298, USA; 5 Department of Anatomy and Neurobiology, Virginia Commonwealth University, Richmond, VA, 23298, USA

## Abstract

**Background:**

Lewy bodies (LB) are a neuropathological hallmark of Parkinson’s disease (PD) and other synucleinopathies. The role their formation plays in disease pathogenesis is not well understood, in part because studies of LB have been limited to examination of post-mortem tissue. LB formation may be detrimental to neuronal survival or merely an adaptive response to other ongoing pathological processes. In a human cytoplasmic hybrid (cybrid) neural cell model that expresses mitochondrial DNA from PD patients, we observed spontaneous formation of intracellular protein aggregates (“cybrid LB” or CLB) that replicate morphological and biochemical properties of native, cortical LB. We studied mitochondrial morphology, bioenergetics and biogenesis signaling by creating stable sub-clones of three PD cybrid cell lines derived from cells expressing CLB.

**Results:**

Cloning based on CLB expression had a differential effect on mitochondrial morphology, movement and oxygen utilization in each of three sub-cloned lines, but no long-term change in CLB expression. In one line (PD63_CLB_), mitochondrial function declined compared to the original PD cybrid line (PD63_Orig_) due to low levels of mtDNA in nucleoids. In another cell line (PD61_Orig_), the reverse was true, and cellular and mitochondrial function improved after sub-cloning for CLB expression (PD61_CLB_). In the third cell line (PD67_Orig_), there was no change in function after selection for CLB expression (PD67_CLB_).

**Conclusions:**

Expression of mitochondrial DNA derived from PD patients in cybrid cell lines induced the spontaneous formation of CLB. The creation of three sub-cloned cybrid lines from cells expressing CLB resulted in differential phenotypic changes in mitochondrial and cellular function. These changes were driven by the expression of patient derived mitochondrial DNA in nucleoids, rather than by the presence of CLB. Our studies suggest that mitochondrial DNA plays an important role in cellular and mitochondrial dysfunction in PD. Additional studies will be needed to assess the direct effect of CLB expression on cellular and mitochondrial function.

## Background

The neuropathological diagnosis of Parkinson’s disease (PD) is based on the loss of dopaminergic neurons in the substantia nigra, as well as by the presence of Lewy bodies (LB) and Lewy neurites in the substantia nigra and other brain regions [1]. A plethora of models have served as the foundation for research into PD pathogenesis. They range from yeast to primates and utilize nuclear gene expression based on inherited forms of PD, as well as dopaminergic neurotoxins [2].

Of these PD models, human cytoplasmic hybrids or “cybrids” are unique because platelet-derived mitochondrial DNA (mtDNA) from sporadic PD patients is expressed in mtDNA-free (Rho0) human neuroblastoma (SH-SY5Y) cells or other cell lines [3,4]. Human cybrid cell lines provide an opportunity to study cellular consequences of the expression of mtDNA from sporadic PD patients.

Recent studies have clearly linked the consequences of mitochondrial dysfunction with sporadic and familial forms of PD [5,6]. Our PD cybrid lines are characterized, in part, by abnormalities in oxygen utilization and mitochondrial electron transport chain (mtETC) function [3,7]. In addition, PD cybrid lines spontaneously generate intracellular proteinaceous aggregates (cybrid Lewy bodies: CLB) that replicate the composition and ultrastructure of cortical LB [8]. Like LB in PD brain sections, CLB in our PD cybrid lines created from individual PD patients stain with eosin, Congo red, Thioflavin S, α-synuclein, and ubiquitin, as well as with markers for mitochondria, the proteasome and lysosomes [8]. Until recently, LB studies have been limited to the analysis of post-mortem tissues [9-11]. CLB formation in PD cybrids provides a unique opportunity to explore the influence of LB formation on cellular and mitochondrial function in a live cell model.

**Figure 1:**
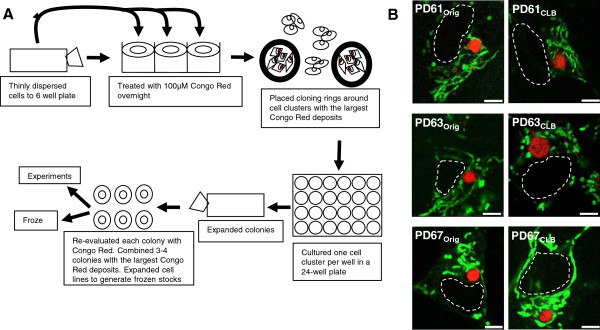
**PD**_**CLB **_**cell lines were created from PD**_**Orig **_**cell lines. (A)** PD_CLB_ lines were created by selecting for cells containing large, circular, Congo red positive inclusions (CLB) as shown in this diagram. **(B)** PD_Orig_ and PD_CLB_ cybrid cells were stained with Congo red to visualize CLB (red) and MitoTrackerGreenFM to visualize mitochondria (green). The nucleus is indicated by white dashed line. Of the three PD_Orig_ and PD_CLB_ pairs, only PD63_CLB_ had mitochondria that were morphologically different from PD63_Orig_. Scale bar = 5μm.

In an effort to better understand the relationship between CLB expression and mitochondrial and cellular dysfunction, we selected three different CLB-expressing PD cybrid cell lines that exhibit a range in oxygen consumption from severely compromised to near normal function. The three PD cybrid lines used in this paper were generated from platelets donated by patients at stage 2.0 Hoehn and Yahr Parkinson’s disease staging score (see Additional file 1). Other patient characteristics such as age, disease duration, L-dopa therapy and presence of dementia were also determined (see Additional file 1). Each of these three original PD cybrid lines (PD61_Orig_, PD63_Orig_, PD67_Orig_) was sub-cloned to enrich for cells expressing CLB by labeling the CLB in living cells with Congo red. Cell clusters expressing CLB from each PD line were selected, sub-cloned and expanded to generate cybrid lines PD61_CLB_, PD63_CLB_ and PD67_CLB_ (see Figure 1A).

Because other investigators have suggested that LB may be detrimental to neuronal function and survival [12-14], we anticipated that CLB-selected PD cybrid lines (PD_CLB_) would exhibit compromised function compared to the original PD cybrid lines (PD_Orig_). Contrary to our expectations, enrichment for CLB expression differentially affected each of the three PD_Orig_ cybrid lines. Cellular and mitochondrial function improved in PD61_CLB_, worsened in PD63_CLB_ and was unchanged in PD67_CLB_. Analysis of our results indicates that CLB expression in PD_CLB_ cybrid lines did not correlate with the changes in cellular and mitochondrial function we detected. Rather, the change in function between PD_Orig_ and PD_CLB_ cybrid lines was determined by the presence or absence of mtDNA in nucleoids in PD_Orig_ cells containing CLB.

## Results and Discussion

### CLB morphology and composition in PD_Orig_ and PD_CLB_ cybrid lines

Like LB in PD brain tissue, CLB in all PD_Orig_ and PD_CLB_ cybrid lines exhibited a consistent range in size (see Additional file 2) and stained uniformly with the histochemical dye Congo red (Figure 1B). Congo red binds to fibrillar α-synuclein as well as other misfolded, amyloidal (beta-pleated sheet folded) proteins [15]. While Congo red does not cross the blood brain barrier, it will cross living cell membranes and label intracellular amyloidal aggregates in vitro [16,17]. Like LB in PD brain, CLB also labeled with antibodies to α-synuclein and polyubiquitin (see Additional file 3).

**Figure 2:**
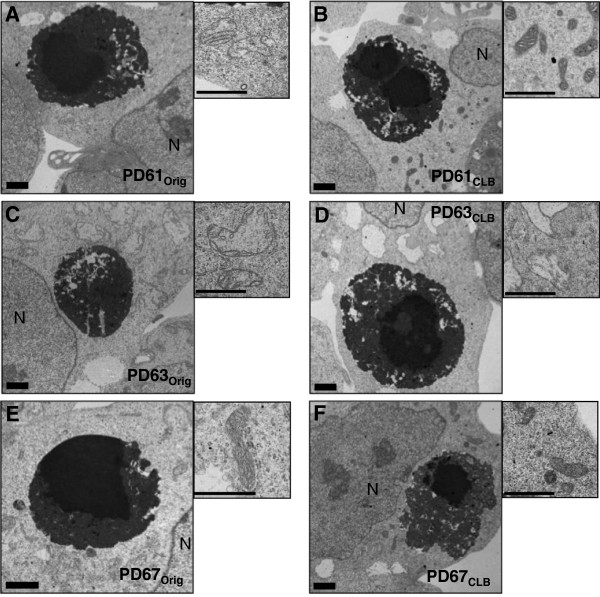
**Electron micrographs of CLB and mitochondria in PD**_**Orig **_**and PD**_**CLB **_**cybrid lines.** CLB in PD_Orig_**(A, C, E)** and PD_CLB_**(B, D, F)** lines typically had an electron dense, compact core (core in PD63_CLB_ is a double). The dense core is surrounded by a halo consisting of electron dense, heterogeneous aggregated material. Paired higher magnification images illustrate mitochondrial morphology in cells containing CLB. **(A, B)** The mitochondria in PD61_Orig_ were enlarged with reduced numbers of cristae and a pale matrix. Mitochondria in PD61_CLB_ were normal in appearance with a dense matrix and regular cristae. **(C, D)** Mitochondria in PD63_CLB_ were swollen with reduced numbers of fragmented cristae and a little to no matrix while mitochondria in PD63Orig were swollen with fragmented cristae and a pale matrix. **(E, F)** Mitochondria in PD67_Orig_ and PD67_CLB_ were rod-like, dense, and featured organized cristae. N= nucleus. Scale bar = 1μm.

Using electron microscopy, CLB in all six PD_Orig_ and PD_CLB_ lines were structurally equivalent (Figure 2). The heterogeneous, dense granular appearance of CLB at the electron microscope level (EM) suggests that small protein aggregates contribute to the continuous formation of CLB (Figure 2). LB in PD brain tissue are also composed of aggregated, dense granular material [18]. CLB do not consistently contain straight filaments, consequently they more closely resemble cortical LB, rather than brainstem LB [8].

### Generation of PD_CLB_ lines using Congo red to select CLB-expressing cells

In cultures of PD61_Orig_, PD63_Orig_ and PD67_Orig_ cybrid lines, cells with Congo red-labeled CLB were selected and propagated to generate sub-cloned lines enriched for cells with CLB (PD61_CLB_, PD63_CLB_ and PD67_CLB_). PD_CLB_ sub-cloned lines were propagated for 30 to 74 days, at which time numerous aliquots of each line were frozen for future use (see Figure 1A and Methods). Early in the sub-cloning process, CLB expression was increased (data not shown). However, assessment of Congo red-positive pixel intensity, pixel length or pixel area in cultures derived from current frozen stocks of PD_CLB_ lines (at least 4-12 passages older) revealed no difference in the frequency of Congo red-positive staining among the PD_Orig_ and PD_CLB_ lines. Furthermore, there was no change in CLB expression frequency (see Additional file 2).

To understand why increased CLB expression was not sustained in PD_CLB_ cybrid lines, it is important to think of CLB as aggresomes (as suggested by Olanow et al. [19]). Aggresomes are created in vitro by transiently inducing protein misfolding or the over-expression of proteins that are prone to misfolding [19]. Because the generation of misfolded and damaged proteins is continuous in PD cybrid lines, we consider CLB to be “professional aggresomes.” Like aggresomes, CLB form in the perinuclear region, contain punctate gamma tubulin staining and are composed of aggregated, damaged and misfolded proteins [8]. Some speculate that LB are “permanent trash dumps”, while others consider LB to be recycling centers [19]. Further studies will be necessary to address this important topic. One way to determine if CLB are trash dumps or recycling centers is to visualize changes in the distribution and expression of fluorescently labeled proteins in CLB using fluorescence recovery after photobleaching.

In PD substantia nigra, the expression level of LB appears to be constant (3-4%) irrespective of the duration of the disease. This observation is consistent with the idea that LB are constantly forming and being eliminated [20]. The frequency of CLB expression in PD_CLB_ clones and parent PD_Orig_ cybrid cell lines was also comparable to the frequency of LB found in PD patient brain [21].

There are several potential mechanisms that could contribute to CLB expression levels in PD cybrid lines. Rujano et al. [22] showed that aggresomes are asymmetrically distributed during somatic and stem cell mitosis. One daughter cell retains the aggresome while the other is free of damaged and misfolded proteins [22,23]. The same asymmetric inheritance of aggresomes also happens when a cybrid cell with a CLB undergoes mitosis (data not shown). If CLB-free daughter cybrid cells proliferate more efficiently than cells with the burden of a CLB, the frequency of CLB-positive cells would decline during cell line expansion after initial sub-cloning. CLB-positive cells are not completely eliminated from cybrid lines because protein misfolding and aggregation is an ongoing process. Each PD_Orig_ and PD_CLB_ cybrid line (see Additional file 2) achieved a steady state level of CLB expression comparable to Rujano et al. [22].

Another potential mechanism that may play a role in the steady state level of CLB expression is cytoplasmic extrusion. Extracellular LB have been identified in PD brain sections using α-synuclein antibodies [24]. Doehner et al. [25] characterized the accumulation of granular Reelin/ β-amyloid deposits in mouse hippocampus. They detected Reelin-positive budd-like extrusions that they claim represent a protective reaction by postmitotic neurons with impaired protein degradation pathways. The extruded misfolded proteins, mitochondria, vacuoles and debris are then cleared by intrinsic glia [25]. Extracellular CLB have been seen in cultures of PD cybrid cell lines (See Additional file 4) that may be the result of cytoplasmic extrusion. The time-lapse studies of CLB-expressing cells needed to confirm this possibility are beyond the scope of this paper.

### Mitochondrial morphology in PD_Orig_ and PD_CLB_ cybrid lines

Mitochondrial shape and changes in shape are intrinsically related to essential cellular functions such as mitochondrial membrane potential, ATP production, calcium signaling and ROS generation (reviewed in [26]). Consequently, the morphology of mitochondria either at the light or EM level provides insight into their functional capacity. Using light microscopy, we observed that mitochondria in PD61_Orig_, PD63_Orig_ and PD67_Orig_ cells containing CLB varied from elongate to short rod-like or globular in shape (Figure 1B). The mitochondrial morphology in the PD_Orig_ lines was consistent with previous studies of PD cybrid cell lines [27].

Mitochondrial morphology at the light microscope level was qualitatively unchanged in PD61_CLB_ and PD67_CLB_ when compared to PD61_Orig_ and PD67_Orig_, respectively (Figure 1B). However the mitochondria in PD63_CLB_ were noticeably different from those in PD63_Orig_ (Figure 1B). PD63_CLB_ mitochondria were swollen, fragmented and globular, rather than rod-like.

The shift from rod-like mitochondria in PD63_Orig_ to swollen, fragmented and globular mitochondria in PD63_CLB_ is evidence of altered mitochondrial dynamics. Mitochondrial fragmentation can have many different causes [28]. Fragmented and dysfunctional, rather than elongated mitochondria, are more susceptible to mitophagy [29,30]. Future studies will be necessary to reveal the specific cause of mitochondrial fragmentation in PD63_CLB_. Mitochondrial movement, especially in neuronal processes, is also influenced by mitochondrial shape (reviewed in [31]). Measurement of mitochondrial movement in the processes of differentiated PD_Orig_ and PD_CLB_ cybrid neurons is described below.

In light of these observations, we also processed fixed pellets of each PD_Orig_ and PD_CLB_ cell line for EM (Figure 2). Mitochondrial morphology at the EM level was qualitatively unchanged in PD67_CLB_ when compared to PD67_Orig_ (Figure 2E, F). The majority of the mitochondria in both PD67_Orig_ as well as PD67_CLB_ cells exhibited normal morphology with a rod-like shape, organized cristae and a dense matrix (Figure 2E, F). At the EM level, mitochondria in PD61_Orig_ were enlarged (increased width) with a pale matrix and reduced numbers of cristae (Figure 2A, B). The mitochondrial morphology in PD61_CLB_ was improved compared to PD61_Orig_ with normal appearing rod-like mitochondria with a dense matrix and intact cristae (Figure 2A, B). The mitochondria in PD63_Orig_ cells were swollen with a pale matrix and reduced and irregularly shaped cristae (Figure 2C, D). PD63_CLB_ had primarily globular mitochondria with few cristae and a transparent matrix (Figure 2C, D).

Many critical mitochondrial functions are localized to cristae, such as the mtETC, iron/sulfur cluster biogenesis and the transport of mtDNA encoded proteins according to Zick et al. [32]. Therefore, the severe loss and disruption of cristae in PD63_Orig_ and PD63_CLB_ suggests that functions such as the mtETC and oxygen utilization should be dysfunctional. It has been estimated that 67% of all mitochondrial proteins are located in the matrix [33]. The matrix is the site for hundreds of enzymes, some of which participate in pyruvate and fatty acid metabolism and the citric acid cycle. Mitochondrial DNA enclosed in nucleoids, mitochondrial ribosomes and tRNAs are also localized in the matrix. The loss of matrix density in globular mitochondria in cybrid cells like PD63_Orig_ and PD63_CLB_ is indicative of a functionally disabled organelle that is a potential risk to the cell it occupies. Changes in mitochondrial morphology, such as the conversion of rod-like shapes to globular shapes can alter the cellular distribution of mitochondria. In complex cells like neurons, swollen and globular mitochondria can contribute to loss of synaptic function or cell death because these morphologically abnormal mitochondria cannot be transported into narrow caliber axons and dendrites [31,34].

### Mitochondrial oxygen consumption in PD_Orig_ and PD_CLB_ lines

**Figure 3:**
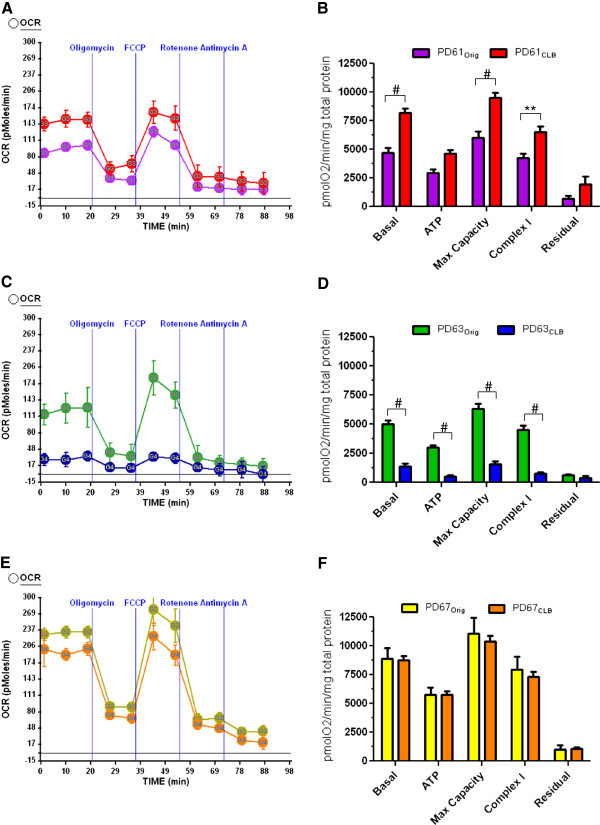
**Respiration rates of PD**_**Orig **_**and PD**_**CLB **_**cybrid clones. (A, C, E)** OCR was measured using the Seahorse XF24 analyzer for all three PD cybrid pairs and controls (not shown). Oligomycin, FCCP, rotenone, and antimycin A were added at the same time point for each experiment. in sequential to measure specific components of the mtETC (see Methods). **(A, B)** PD61_CLB_ had significantly higher OCR but not residual (non-mitochondrial) respiration than PD61_Orig_ (n=8). **(C, D)** PD63_CLB_ had significantly lower OCR than PD63_Orig_ (n=10). **(E, F)** OCR in PD67_CLB_ did not differ significantly from PD67_Orig_ (n=10). Two-way ANOVA with Bonferroni multiple comparisons; *, p<0.05; **, p<0.01; #, p<0.001.

Taking into account the abnormalities in mitochondrial morphology between some PD_Orig_ and PD_CLB_ cybrid lines, we measured oxygen consumption using a Seahorse Extracellular Flux Analyzer XF24 (Seahorse Bioscience) [35-37]. The three PD_Orig_ lines that were selected for CLB cloning expressed a range of basal oxygen consumption values prior to cloning (Figure 3). If CLB expression is detrimental to cell function, then we anticipated that all three PD_CLB_ lines would exhibit reduced oxygen consumption compared to PD_Orig_ lines. Figure 3 shows the oxygen consumption rates (OCR) of confluent cultures of PD_Orig_ and PD_CLB_ lines at baseline and after sequential treatment with specific inhibitors (oligomycin to inhibit ATP synthase, carbonyl cyanide 4-(trifluoromethoxy)phenylhydrazone (FCCP) to dissipate the proton gradient across the inner mitochondrial membrane, rotenone to inhibit complex I and antimycin A to inhibit complex III, see Methods). Use of these inhibitors permits the determination of key aspects of mitochondrial function including basal OCR, maximum capacity OCR, ATP-linked OCR, complex I-linked OCR and the non-mitochondrial (residual) OCR [36,37]. Given the abnormal mitochondrial morphology shown above, it is important to determine if exposure to specific mitochondrial inhibitors during measurements of OCR cause any cell loss. For all three pairs, there was no difference between PD_Orig_ and PD_CLB_ lines in cell viability at the end of the experiment, as measured by live-dead counts (data not shown). The cell viability in these cell lines also did not significantly differ from the disease-free controls (n= three control lines).

PD61_CLB_ had significantly higher basal, maximal and complex I-linked OCR when compared to PD61_Orig_ (Figure 3A, B) suggesting that sub-cloning of cells expressing CLB resulted in improved oxygen consumption in PD61_CLB_ cells. This change was mtETC selective because there was no significant change in glycolysis (measured as extracellular acidification rate- ECAR, a surrogate for lactate production and aerobic glycolysis, data not shown) or in non-mitochondrial (residual) respiration. Significant improvements in basal, complex I-linked and maximum capacity OCR are also consistent with the improvement in mitochondrial ultrastructure in PD61_CLB_ cells compared to PD61_Orig_ (Figure 2B).

In contrast, PD63_CLB_ had minimal levels of basal OCR and its response to mitochondrial inhibitors was significantly reduced when compared with PD63_Orig_ (Figure 3C, D). There were also significant reductions in basal, maximum capacity, complex I- and ATP-linked OCR in PD63_CLB_ compared to PD63_Orig_. However, there was no significant change in non-mitochondrial respiration. Therefore, this change in OCR in PD63_CLB_ was selective for mtETC. There was also no compensatory up-regulation in ECAR in PD63_CLB_. This was surprising because other studies have shown that loss of complex I activity as a result of neurotoxicity induces a loss of OCR with a corresponding increase in ECAR [38]. This loss of mtETC function and OCR is consistent with the abnormal morphology of mitochondria in PD63_CLB_ cells (Figure 2D).

Finally, PD67_CLB_ exhibited basal, maximum capacity and complex I-linked OCR that was unchanged from PD67_Orig_ (Figure 3E, F). There was also no change in ECAR (glycolysis) or non-mitochondrial respiration. These OCR values correlate with the consistent, normal morphology of mitochondria in PD67_Orig_ and PD67_CLB_ at the light and EM levels (Figures 1B, 2E, F).

### Movement of mitochondria by axonal transport in PD_Orig_ and PD_CLB_ cybrid lines

One hypothesis for the dopaminergic neuron terminal degeneration seen in patients with sporadic PD is axonal transport failure [39]. Reduced axonal transport deprives the cell body of vital trophic factors and deprives axon terminals of synaptic vesicles and organelles like mitochondria [40]. Proper distribution of mitochondria to synapses is also crucial for synaptic homeostasis in response to changes in synaptic activity (reviewed by [41]). Based on studies of post-mortem sections of PD brain, Kanazawa et al. [42] concluded that LB and Lewy neurites are involved in altered axonal transport because LB can become Lewy neurites. Mitochondrial movement both anterograde and retrograde depends on motor proteins that utilize ATP [43]. Chu et al. [39] reported a decline in motor proteins early in sporadic PD brain that precedes other PD related changes like loss of dopamine or tyrosine hydroxylase. This loss of motor protein expression was also highest in nigral neurons containing α-synuclein inclusions.

**Figure 4:**
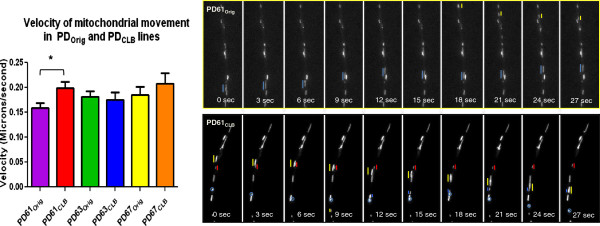
**Mitochondrial movement in differentiated in PD**_**Orig **_**and PD**_**CLB **_**cell lines.** The graph shows the velocity of mitochondrial movement in processes of differentiated PD61_Orig_ (n=4 cultures, 73 processes), PD61_CLB_ (n=4 cultures, 94 processes), PD67_Orig_ (n=4 cultures, 98 processes), PD67_CLB_ (n=5 cultures, 108 processes), PD63_Orig_ (n=8 cultures, 167 processes) and PD63_CLB_ (n=4 cultures, 80 processes). The velocity of mitochondrial movement was significantly higher in PD61_CLB_ when compared with PD61_Orig_ determined by a *t*-test assuming equal variance. *p<0.03 1-tail and p<0.05 2-tail. The velocity of mitochondrial movement in SH-SY5Y neuronal processes was 0.220 microns per second +/- 0.01S.E.M. (n=3 cultures) and 0.236 microns per second +/-0.017S.E.M. In neuronal processes from three control cybrids (CNTL56, n=2 cultures, CNTL 64, n=3 cultures, CNTL68, n=3 cultures). Two montages illustrate the movement of mitochondria stained with MTRed during a 27sec period. Moving mitochondria in PD61_Orig_ are marked with yellow and blue lines. Moving mitochondria in PD61_CLB_ are marked with yellow, blue and red lines and a blue circle.

We previously showed that axonal transport of mitochondria was significantly reduced in the tyrosine hydroxylase-containing processes of PD cybrids [44]. To study changes in axonal transport, PD_Orig_ and PD_CLB_ cybrid lines were differentiated into neuronal cells using low doses of staurosporine [45]. We measured the axonal transport velocity of fluorescently labeled mitochondria in individual cybrid neuron processes. In agreement with previous studies, mitochondrial velocity was reduced in all differentiated PD_Orig_ cell lines compared to differentiated SH-SY5Y cells [44]. The velocity of mitochondrial movement in PD67_CLB_ was not significantly different from PD67_Orig_ (Figure 4). This outcome is consistent with the lack of change in mitochondrial morphology and OCR in PD67_CLB_ after subcloning. Only PD61_CLB_ exhibited a significant increase in mitochondrial velocity compared to PD61_Orig_ (Figure 4). This result is consistent with other improvements in mitochondrial morphology and OCR (Figures 2B, 3A, B).

Given the poor mitochondrial morphology and OCR in PD63_CLB_, it was a surprise that mitochondrial velocity was not comparably reduced (Figure 4). Whether induced by low dose staurosporine or retinoic acid, neuronal differentiation imposes increased demands for ATP and increased cellular stress. It is likely that cells with poor oxygen utilization and mitochondrial function are incapable of completing the process of differentiation. Therefore the most dysfunctional cells in PD63_CLB_ should fail to differentiate. Additional file 5A depicts typical phase contrast images of PD_Orig_ and PD_CLB_ cybrid cells after differentiation. The cell density in a microscope field of differentiated PD63_Orig_ and PD63_CLB_ cybrid neurons was noticeably lower than the cell density for other cybrid lines. This information is also presented graphically in Additional file 5B. These data support the proposition that PD63_orig_ cells with inadequate mitochondrial function were unable to differentiate and only those cells with sufficient mitochondrial function differentiated into neurons. Consequently the velocity of mitochondrial movement in differentiated PD63_CLB_ neurons was comparable to other PD cybrid cell lines.

### Nucleoid density in PD_Orig_ and PD_CLB_ cybrid cell lines

**Figure 5:**
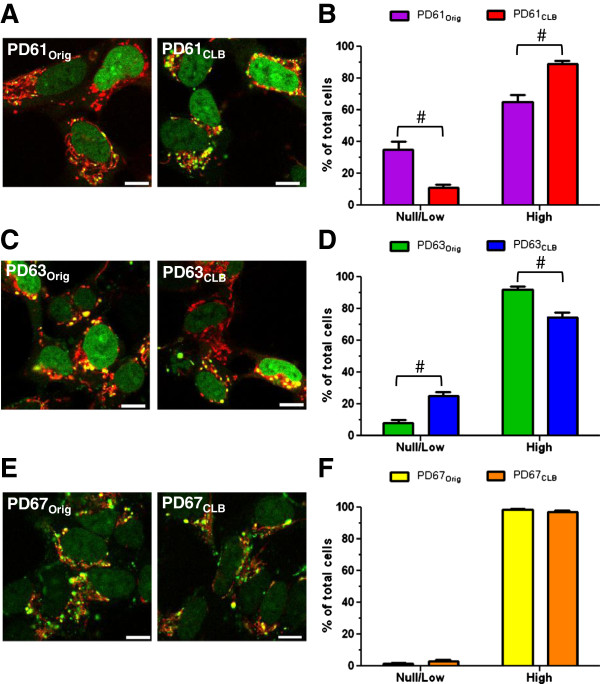
**Nucleoid content in PD**_**Orig **_**and PD**_**CLB **_**cybrid lines. (A, C, E)** Representative images of nucleoids in the PD_Orig_ and PD_CLB_ pairs using PicoGreen and MTRed (green: DNA, red: mitochondria). Yellow puncta are nucleoids in mitochondria. Cells that lack nucleoids are red with no yellow puncta (**A**). The nucleoids in PD63_Orig_ and PD63_CLB_**(C)** were larger in diameter. **(B, D, F**) Cells were scored for nucleoid content as either “null/low” or “high” (see Methods) and graphed as a percentage of total cells, n= 5; #, p<0.001. **(A, B)** Consistent with other data, PD61_CLB_ showed a significant increase in percent of cells that scored “high” and had fewer cells that scored “null/low” for nucleoid content compared to PD61_Orig_. **(C,D)** Conversely, PD63_CLB_ showed an increase in cells that scored “null/low” and decrease in cells with “high” nucleoid density compared to PD63_Orig_. **(E,F)** There was no difference in nucleoid content between PD67_CLB_ and PD67_Orig_. Two-way ANOVA with Bonferroni multiple comparisons, n = 5; #, p<0.001. Scale bar=5μm.

The sub-cloning of cybrid cells expressing CLB did not result in a uniform change in mitochondrial function among the PD cybrid cell line pairs. To establish if changes resulting from sub-cloning could be due to changes in mtDNA distribution, we first visualized nucleoids. Nucleoids are structures consisting of one or more mtDNA molecules and associated proteins like single-stranded DNA binding protein, Twinkle, mtDNA helicase and mitochondrial transcription factor A (TFAM) among others [46-48]. To visualize nucleoids, we used the DNA stain PicoGreen in combination with MitoTracker CMXRos (MTRed; Figure 5A, C, E) in live PD_Orig_ and PD_CLB_ cybrid cells. Nucleoid content was scored as “low/null” or “high” in cells from each of the PD cybrid pairs (Figure 5B, D, F, see Methods). Rho0 cells that lack mtDNA are devoid of PicoGreen staining and nucleoids (data not shown and [46]). PicoGreen staining is also independent of membrane potential or mtETC function [46]. PD61_Orig_ contained cells that fell into the “low/null” category as well as cells with “high” numbers of nucleoids (Figure 5A, B). In contrast, PD61_CLB_ contained significantly fewer cells in the “low/null” category and more cells in the “high” nucleoid category compared to PD61_Orig_ (Figure 5B). This increase in cells with “high” numbers of nucleoids is consistent with previous data in this paper showing an improvement in mitochondrial function and morphology in PD61_CLB_. PD63_CLB_ had significantly more cells that scored “low/null” and fewer cells in the “high” category than PD63_Orig_ (Figure 5D). This was not surprising given the decline in PD63_CLB_ mitochondrial function and morphology. Levels of nucleoid expression in PD67_Orig_ and PD67_CLB_ were comparable and consistent with previous data in this paper (Figure 5F).

**Figure 6:**
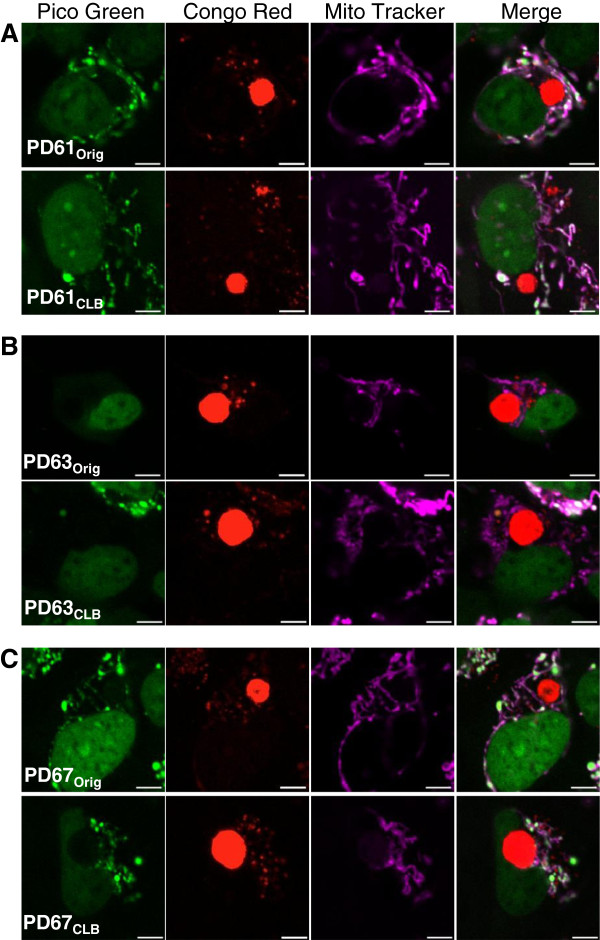
**Nucleoid content in cells containing CLB in PD**_**Orig **_**and PD**_**CLB **_**cell lines. (A,B,C)** Representative images of cells containing CLB in PD_Orig_ and PD_CLB_ pairs. Cells were triple-stained with PicoGreen, Congo red, and MitoTracker Deep Red (green: DNA, red: CLB, purple: mitochondria). **(A)** CLB-containing cell in PD61_Orig_ contained nucleoids that co-localized with mitochondria. PD61_CLB_ (bottom panel) also featured CLB-containing cells with contained nucleoids. **(B)** CLB-containing cells from PD63_Orig_ (top panel) and PD63_CLB_ (bottom panel) did not contain nucleoids. **(C)** Cells containing CLB in PD67_Orig_ (top panel) and PD67_CLB_ (bottom panel) contained nucleoids. Scale bar=5μm-.

In light of these results, we visualized nucleoids in individual PD_Orig_ and PD_CLB_ cells containing CLB using fluorescent markers: Congo red (CLB and small protein aggregates), PicoGreen (nucleoids) and MitoTracker Deep Red (mitochondria) as shown in Figure 6. Remarkably, we found that all CLB-positive PD61_Orig_ cells contained numerous nucleoids (Figure 6A, top panel). The same result was true of PD67_Orig_ (Figure 6C, top panel). However, the majority of CLB-expressing cells in PD63_Orig_ did not contain nucleoids (Figure 6B, top panel). Taken together, these results indicate that the nucleoid content of the PD_Orig_ cells containing CLB correlates with changes in mitochondrial quality and function detected in PD_CLB_ cell lines. PD61_CLB_ had better mitochondrial quality and function because it was sub-cloned from PD61_Orig_ CLB-expressing cells containing numerous nucleoids. PD63_CLB_ had reduced mitochondrial quality and function because it was sub-cloned from CLB-containing cells in PD63_Orig_ with few nucleoids. CLB-containing cells in PD67_Orig_ had numerous nucleoids and these cells yielded the PD67_CLB_ cybrid line that also had cells with high numbers of nucleoids as well as adequate mitochondrial quality and function.

### Gene expression levels for mtETC genes in PD_Orig_ and PD_CLB_ cell lines

**Figure 7:**
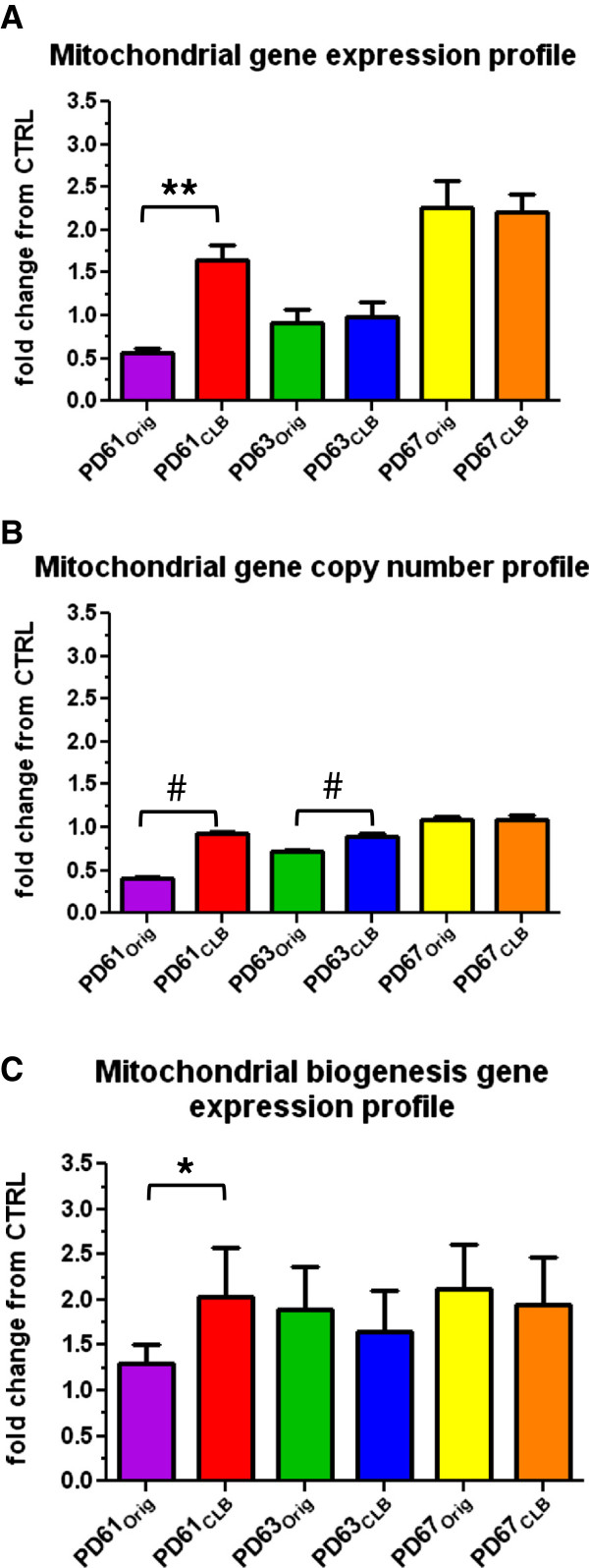
**Quantitative real-time PCR analysis of gene profiles in PD**_**Orig **_**and PD**_**CLB **_**cell lines. (A)** Mitochondrial gene expression for four mtDNA encoded genes (ND2, CO3, ND4, 12srRNA) measured using RT-qPCR from cDNA and compiled to create a gene expression profile. Starting quantities were normalized to the geometric mean for each cell line and graphed as the average fold change from the mean from three CNTL cybrid lines (56, 64, 68) for each gene in this profile. **(B)** Mitochondrial gene copy numbers for the same four genes was assayed from gDNA and expressed as described above. **(C)** Mitochondrial biogenesis gene expression (PGC-1α, NRF1, TFAM, TFB1M) was measured from cDNA and expressed as described above. Student’s *t*-test, with Welch’s correction in the case of non-equal variances; *, p<0.05; **, p<0.01; #, p<0.001.

Since nucleoids contain mtDNA, we examined mtDNA copy number and expression in PD_Orig_ and PD_CLB_ lines. Previous studies have shown that complex I is damaged and functionally impaired in post-mortem PD cortex homogenates [49]. Analysis of PD cybrid cell lines (including the three PD_Orig_ lines included in the paper) showed that complex I gene expression was reduced and showed a robust correlation with the changes in mtETC gene expression found in post-mortem PD cortex [50]. Enzymatic dysfunction related to complex I assembly is often associated with deficiencies in complexes III and IV because complex I assembly intermediates act like a scaffold for the assembly of other complexes in the mtETC [51]. We therefore measured gene expression and gene copy number for mitochondrial genes ND2 and ND4 (complex I), CO3 (complex IV) and 12s ribosomal RNA using quantitative real-time polymerase chain reaction (RT-qPCR) to create mitochondrial gene expression and copy number profiles (Figure 7).

Figure 7A and B show that mitochondrial gene expression in PD61_CLB_ increased nearly 3-fold and copy number increased more than 2-fold over PD61_Orig_. This observed improvement in mitochondrial gene expression and copy number in PD61_CLB_ is consistent with the improved nucleoid content in PD61_CLB_ cells. Furthermore, mtDNA copy number improved to control levels in PD61_CLB_ while mtDNA expression levels were more than 2-fold higher than control levels. These results also suggest a potential mechanism for the improved mitochondrial morphology, mitochondrial respiration and mitochondrial movement observed in PD61_CLB_ (Figures 1B, 2A, 2B, 3A, 3B, 4A) and supports our conclusion that mtDNA content in the cells containing CLB in PD61_Orig_ was a driving factor in the functional improvements we observed in PD61_CLB_.

Mitochondrial gene expression was unchanged and remained reduced, as compared to control in both PD63_Orig_ and PD63_CLB_ (Figure 7A). There was a slight but significant increase (less than 2-fold) in mitochondrial gene copy number in PD63_CLB_ (Figure 7B). This increase in gene copy number was surprising. We expected a decrease in mitochondrial gene copy number considering the reduced number of cells that scored “high” for nucleoid content (Figure 5C, D). Ashley et al. [46] suggested that fusion of nucleoids can occur as cells strive to maintain mtDNA copy number. Also, there is a linear relationship between mtDNA content and nucleoid volume [52]. Consequently, the large nucleoids in PD63_CLB_ (Figure 5C) may harbor increased numbers of mtDNA. Additionally, PD63_CLB_ had a decline in mitochondrial function and decrease in mitochondrial respiration, as compared with PD63_Orig_ (Figures 1B, 2C, 2D; 3C, 3D, 4A). We speculate that this slight increase in mitochondrial gene copy number could be a compensatory mechanism. Since there was no change in mitochondrial gene expression, the increase in mtDNA copy number did not have downstream functional consequences. The poor cellular and mitochondrial function in PD63_CLB_ reflects this outcome.

As expected, there was no change in mitochondrial gene expression or copy number between PD67_Orig_ and PD67_CLB_ (Figure 7A, B). Both of these cell lines exhibited similar mitochondrial morphology, mitochondrial respiration, mitochondrial movement and nucleoid content (Figures 1, 2, 3, 4). These results suggest that creation of PD67_CLB_ from PD67_Orig_ cells containing CLB did not substantially alter mtDNA genetic or phenotypic expression.

### Biogenesis gene expression in PD_Orig_ and PD_CLB_ lines

Cellular regulation of mitochondrial biogenesis is critical for the maintenance of a functional pool of mitochondria in neurons [53]. In fact, the mitochondrial biogenesis pathway has emerged as a potential therapeutic target for PD [53,54]. Peroxisome proliferator-activated receptor gamma co-activator 1-alpha (PGC-1α) is a transcriptional co-activator and serves as the master regulator of mitochondrial biogenesis (reviewed by [55]). A genome-wide analysis of PD patients and controls found that PGC-1α expression was reduced in PD patients [54]. Overexpression of PGC-1α in neurons was found to be protective in a neurotoxin mouse model of PD [56]. In cell culture, primary fibroblasts and cybrids generated using mtDNA from patients with mitochondrial diseases showed improved respiration after increased PGC1-α expression [57]. PPAR (peroxisome proliferator-activated receptor) agonists, such as bezafibrate (an agonist of PPARα), have also been shown to improve mitochondrial function in patient fibroblasts and myoblasts [58] and in a mouse model of mitochondrial disease [59].

To explore what role mitochondrial biogenesis plays in the mitochondrial changes found between the PD_Orig_ and PD_CLB_ lines, we measured the expression of four mitochondrial biogenesis genes including PGC-1α, nuclear respiratory factor 1 (NRF1), mitochondrial transcription factor B1 (TFB1M) and mitochondrial transcription factor A (TFAM), and used their expression levels to create a mitochondrial gene biogenesis profile. NRF1 is a DNA-binding protein that serves to positively regulate nuclear-encoded subunits of the mtETC [60]. In contrast, TFB1M and TFAM bind directly to mtDNA to initiate mitochondrial-encoded gene transcription [61,62]. These four genes represent control of nuclear- and mitochondrial-encoded mtETC gene transcription, thereby creating a gene expression profile that can be used to quantitate cell-wide activation of mitochondrial biogenesis.

In PD61_CLB_, expression of mitochondrial biogenesis genes was significantly increased by approximately 1.5-fold compared to PD61_Orig_ (Figure 7C). Improved biogenesis is consistent with the increased mitochondrial gene expression and mtDNA copy number described above (Figure 7A, B) and with the general improvement in cellular and mitochondrial function in PD61_CLB_ compared to PD61_Orig_. This improvement in biogenesis may represent a shift in the population of cybrid cells in PD61_CLB_ to include more cells with improved mtDNA gene expression and copy number, or it may represent the improved expression of mitochondrial genes within cells. PGC-1α enhances mtETC function and biogenesis by integrating cellular signals such as AMP/ATP ratios via the AMP-activated kinase (reviewed in [63]). Further research into this area would be beneficial for understanding the therapeutic potential of PGC-1α manipulation.

There was no difference in biogenesis gene expression between PD63_Orig_ and PD63_CLB_ (Figure 7C). This was not unexpected due to the decline in mitochondrial function in PD63_CLB_, compared to PD63_Orig_ (Figures 3, 4). There was also no change in biogenesis gene expression between PD67_Orig_ and PD67_CLB_ (Figure 7C). This was also expected because there was no difference in mitochondrial gene expression, gene copy number, or mitochondrial function between these two cell lines.

It is important to remember that in the cybrid model, mtDNA from individual patients is expressed against a common background of nuclear genes from the SH-SY5Y parent cell line. The differences we observed in expression of nuclear mitochondrial biogenesis genes across different cybrid lines are ultimately a consequence of the presence of individual patients’ mtDNA. However, the differences in mitochondrial biogenesis signaling across PD_CLB_ compared to PD_Orig_ cell lines within each cybrid line are derived from selection of CLB-producing cybrids. We found that selection of cybrid cells for CLB expression had a differential effect on mitochondrial biogenesis. Mitochondrial biogenesis gene expression improved in PD61_CLB_, but was unchanged in PD63_CLB_ and in PD67_CLB_. The molecular origins of these different consequences of CLB cloning remain unclear but suggest that the correlation between CLB expression and mitochondrial biogenesis signaling is not consistent.

### Concluding Remarks

Due to the lack of PD models that spontaneously make LB, studies have been limited to cataloging contents or inferring mechanism of formation and cellular consequences of LB formation from post-mortem tissue (for example, [9-11]). While neuropathological studies have been very valuable, a live cell model would help investigate the biological significance of LB for PD pathogenesis. The discovery that PD cybrid cell lines spontaneously form and express CLB has provided us with a much needed live cell model of LB. CLB display the components found in LB in PD brain including eosinophilia, α-synuclein-, ubiquitin-, parkin- and Thioflavin S-staining [8]. Furthermore, CLB in PD cybrid lines are generated without the need for genetic over-expression of molecules like α-synuclein or inhibiting proteolytic processes such as proteosomal degradation or autophagy. PD_Orig_ cybrid lines demonstrate that expression of the PD patient platelet mtDNA genes is responsible for the formation of CLB. By generating PD_CLB_ cybrid cell lines from PD_Orig_ cybrid cells expressing CLB, our aim was to create a model that focused on the cellular consequences of CLB formation.

The clinical significance of LB has been widely debated [64]. LB expression in the basolateral nucleus has been associated with visual hallucinations in PD [65]. Also, there are increased numbers of LB in demented versus non-demented PD brain sections from cortex, limbic structures and amygdala [66]. Several research groups have argued that LB are detrimental and contribute to neuronal degeneration in PD [12-14]. Harrower et al. [67], in contrast, proposed that LB mark a “struggling cell,” a concept of the LB that is consistent with the results presented in this paper. Harrower et al. [68] and others have suggested that LB are formed by neurons in a effort to maintain normal function in the face of an ongoing pathological process [11,69-72]. The nature of the pathological process that results in LB formation has also been a subject of speculation. Zhou et al. [73] and Lin et al. [74] suggested that mitochondrial dysfunction precedes and drives LB pathology and neurological dysfunction in PD. The results presented in this paper support the proposal that mitochondrial dysfunction drives CLB pathology. Other alterations in cellular functions such as over-expression and aggregation of mutated α-synuclein or over-expression of wild type α-synuclein can also generate LB [73,75].

If CLB are detrimental for cells, then all three PD_CLB_ lines should have exhibited worsening of cellular and mitochondrial functions. This did occur in PD63_CLB_, but not in PD61_CLB_ or PD67_CLB_. If CLB are an asset with beneficial functions, then cellular and mitochondrial function should improve. This was the outcome in PD61_CLB_ but not in PD63_CLB_ or PD67_CLB_. Sub-cloning CLB-expressing cells to produce PD67_CLB_ did not substantially alter cellular or mitochondrial function. These data suggest that selection and sub-cloning of PD_Orig_ lines for CLB expression did not drive the dysfunction in PD_CLB_ cybrid lines. While α-synuclein aggregation does occur in PD_Orig_ and PD_CLB_ cybrids, levels of α-synuclein expression measured by RT-qPCR were not significantly different between PD_Orig_ and PD_CLB_ lines (see Additional file 6). Further studies are needed to determine what role LB play in PD. As proposed by Kanazawa et al. [42], LB and Lewy neurites may be more specifically involved in the disruption of axonal transport and the removal of damaged and misfolded proteins.

Our studies of PD_Orig_ and PD_CLB_ cybrid cell lines suggest that mtDNA is key to the expression of cellular and mitochondrial dysfunction, such as altered ETC activity and oxygen utilization, abnormal mitochondrial morphology, changes in axonal transport, etc. The PD_CLB_ cell lines that featured significant changes in mitochondrial function and morphology also had distinct changes in mtDNA copy number and/or expression from the PD_Orig_ lines. Our data supports the proposition by Esteves et al. [6] that mtDNA dysfunction is at least partly responsible for mtETC defects in sporadic PD. Exner et al. [5] further concluded that mitochondrial dysfunction is a “common denominator” in the pathogenesis of sporadic and familial PD.

One intriguing finding in our data was the identification of individual cells with CLB that appeared to lack nucleoids and functional mtDNA (see Figure 6B). Since formation of an aggresome or CLB is ATP-driven, it seems unlikely that a cell without mtDNA could generate a CLB. This idea leads to speculation that loss of mtDNA and nucleoids could be part of PD pathogenesis. CLB-bearing PD cybrid cells without mtDNA or nucleoids can survive in culture because of the supportive culture conditions. Nucleoid-free and mtDNA-free neurons are unlikely to survive in vivo unless they can derive sufficient support from surrounding glia. The composition of LB in neuropathological tissue from pre-PD substantia nigra suggests the convergence of multiple pathways such as mitochondrial dysfunction, oxidative stress, oxidative protein damage and altered post-translational modification play a role in PD disease progression [76]. This concept is supported by a recent publication that showed nuclear α-synuclein binds to the PGC-1α promoter in vivo and in vitro, and alters mtDNA copy number and function [77]. Our contribution to this ever-changing field has been to demonstrate the important role that mitochondrial quality, dynamics and function play in PD.

## Methods

### Cybrid cell lines

Cybrid cell lines were created from individual patients and controls as described previously [3,78]. Cells were grown in growth media (GM) consisting of high glucose Dulbecco’s modified Eagle medium (DMEM, Gibco, Life Technologies) with 10% fetal bovine serum, antibiotic/antimycotic, 100μg/ml sodium pyruvate and 50μg/ml uridine, as described previously, to support the survival of cells with mitochondrial dysfunction [8,79]. For imaging, cells were plated on 35mm poly-lysine coated dishes (MatTek Corp.). Cell lines were only kept in culture for a maximum of two months. Cell pellets from PD_Orig_ and PD_CLB_ pairs were always thawed simultaneously and grown under the same culture conditions.

### Generation of sub-cloned cybrid lines based on CLB expression

Glass-bottomed 6-well plates (MatTek Corp.) were treated with 200μg/ml poly-l-lysine/H_2_O (m.w. 30,000-70,000, Sigma-Aldrich) at room temperature for ~40 min. Wells were rinsed twice with sterile water and plates were stored dry until they were loaded with cell suspension (typically 20,000 cells). Selected cybrid lines were harvested from T75 CellStar flasks (Greiner bio-one) with 0.05% trypsin diluted in phosphate buffered saline. GM was used to quench trypsin activity prior to re-plating of cells into glass-bottomed 6-well plates. Cells were incubated at 37°C with 5% CO_2_ for 24-48 hours until cells divided into 2-4 cell clusters. Following an overnight (~18-24 hours) treatment with 100μM Congo red (Sigma-Aldrich) made up in GM, dishes were rinsed twice with GM without phenol red (clearGM) and further stained with 80nM Mitofluor Green (Invitrogen) in clearGM for 20 minutes at 37°C. Wells were again rinsed with clearGM and labeled cells were visualized with epi-fluorescence (Olympus IX-70 microscope) using fluorescein isothiocyanate (FITC) filters (Mitofluor Green) and Texas Red filters (Congo red). Clusters of cells with large (3-5μM) Congo red stained spheres were marked with an inked objective marker (Olympus) on the underside of the coverslip well. After removing GM from the wells, 6mM sterile glass cloning rings were seated around the marked cell clusters using sterile silicone grease (both from Thermo Fisher). Each cell cluster was trypsinized (see above) and re-plated into one well of a 24-well plate. The expression of Congo red positive CLB was later reassessed and the wells with the largest and most numerous Congo red stained spheres were retained and combined (2-5 clones per well). The other clones were discarded. Combined clones were cultured in GM and passed into larger wells as they became confluent. Sub-cloned cybrid lines were expanded into T25 flasks (Greiner bio-one). Each cybrid line was harvested and re-plated into coverslip-bottom dishes and re-selected for CLB expression using Congo red and MitoFluor Green. These colonies were expanded into T75 flasks, at which time aliquots from each line were frozen for subsequent study.

### Quantification of Congo red positive fluorescence

Cells were plated in 35mm dishes as described above and grown for 2-4 days until at least 75% confluent. Cells were stained with Congo red at 100μM for 24 hours. Dishes were then washed two times with clearGM with 25mM Hepes (Gibco, Life Technologies). Dishes were blinded for image collection and quantification. Images were acquired using an Olympus FV1000 confocal microscope (60X objective) at room temperature. Ten representative fields were taken at random per dish and analyzed using MetaMorph image analysis software (Molecular Devices). Studies were repeated with cells from a different passage. Images were set to a common inclusive threshold and pixels over 1μm were measured for total pixel area, pixel intensity and pixel length. Pixel values were normalized to number of cells in each image. To calculate CLB frequency, Congo red positive inclusions over 1μm in diameter were counted for each set of ten images per dish. Number of CLB per dish was normalized to number of cells counted per dish. Student’s t-tests were run to compare the original and sub-clone pairs (Prism, Graph Pad).

### Electron Microscopy

Sub-confluent T75 flasks for each cybrid line were fixed with 2% paraformaldehyde and 2.5% glutaraldehyde in 0.1M phosphate buffered saline, processed for EM, sectioned and stained by staff members of the Advanced Microscopy Facility at the University of Virginia, as previously described [27]. Stained sections were imaged on a Jeol JEM-1230 transmission electron microscope at the Virginia Commonwealth University Microscopy Facility.

### Nucleoid imaging and quantitation

Live cells were grown in 35mm dishes, stained with Quant-It PicoGreen dsDNA and MitoTracker Red CMXRos (both from Molecular Probes/Life Technologies) and imaged in clearGM as described above. Dishes were blinded prior to imaging. Six images were acquired at random per dish using an Olympus FV300 confocal microscope. Cell count totals were acquired by counting the PicoGreen positive nuclei per frame. Nucleoids were defined as areas of PicoGreen and Mitotracker Red colocalization. Cells with less than five nucleoids per cell were considered “low/null”. All others were considered “high”. Only cells with both mitochondria and a nucleus in focus were counted. Two-way ANOVA with Bonferroni multiple comparisons were run to compare PD_Orig_ and PD_CLB_ cell lines at “low/null” versus “high” (Prism, Graph Pad). For analysis of nucleoids in cells with CLB, cells were co-stained with Congo red, MitoTracker Deep Red (50nM for 45 minutes, Molecular Probes/Life Technologies), and PicoGreen. Cells were imaged in clearGM on an Olympus FV1000 confocal microscope.

### Cellular Respiration

Oxygen consumption was measured using the Seahorse Extracellular Flux Analyzer (Seahorse XF24, Seahorse Biosciences) according to manufacturer’s instructions. In brief, cells were plated in Seahorse XF24 culture plates and grown for 24 hours to form a confluent monolayer. One hour prior to each experiment, growth media was exchanged for unbuffered DMEM, pH 7.4. The following inhibitors were used to obtain a bioenergetic profile: oligomycin (1μM), FCCP (300nM), rotenone (100nM), and antimycin A (10μM). For all inhibitors, the pH was adjusted to 7.4 prior to the experiment. For each Seahorse experiment, three basal measurements of the oxygen consumption rate (OCR) were acquired and calculated by the Seahorse XF. Compounds were added in the order mentioned previously, with two measurements following each inhibitor. At the end of each experiment, OCR values were normalized to protein content (Micro BCA Kit, Pierce). OCR values are reported as means ± SEM, except for uncoupled respiration (FCCP), where the highest value was used. Statistics were calculated using two-way ANOVA with Bonferroni multiple comparisons post-hoc tests in Prism software (GraphPad, Prism).

### RT-qPCR

RNA and DNA were extracted from three independent PD_Orig_ and PD_CLB_ cell pellets from sub-confluent T175 flasks using the All Prep RNA/DNA Kit (Qiagen). RNA and genomic DNA (gDNA) were quantified using the NanoDrop 2000 (ThermoScientific). Complimentary DNA (cDNA) was made from RNA using the iScript cDNA Synthesis Kit (Bio-Rad). Quantitative real-time PCR (RT-qPCR) was run on cDNA and gDNA samples to measure mitochondrial gene and mitochondrial biogenesis gene expression, as well as mitochondrial gene copy number. Glyceraldehyde 3-phosphate dehydrogenase, beta-actin and 18sRNA were used as endogenous reference genes. Primer and probe sequences can be found in the Additional file 7. Starting quantities were calculated by Bio-Rad CFX manager software based on cycle threshold of known standards (human fetal brain cDNA, human gDNA or human mtDNA). Samples and standards were run in quadruplicate. All values were normalized to the geometric mean of each sample from the endogenous reference genes. PD_Orig_ and PD_CLB_ data was expressed as a fold change from the pooled mean of three age-matched disease-free control cell lines (Controls 56, 64 and 68) for each gene (see Keeney et al. [7]). The fold changes for each gene from the mean of each individual PD_Orig_ or PD_CLB_ cell line were then averaged to represent the fold increase or decrease from control across each gene profile. Mitochondrial cDNA gene expression and gDNA copy number profiles were made up of ND2, CO3, ND4 and 12srRNA. The mitochondrial biogenesis gene expression profile was made up of PGC-1α, TFAM, NRF1 and TFB1M. Statistical analysis was done using Prism software (GraphPad) using student *t*-test. In the cases of unequal variances, Welch’s correction was performed.

### Axonal transport of mitochondria in the processes of differentiated PD_Orig_ and PD_CLB_ cybrid neurons

Proliferating PD_Orig_ and PD_CLB_ cells were harvested from T75 flasks with 0.05% trypsin (Invitrogen, Life technologies) as previously described [44]. 40,000 cells in 2ml GM were added to each #0 glass bottomed 35mm dishes (MatTek Corp). After 18-24 hours, GM was removed and the differentiation media (DM) consisting of 500ml of Neurobasal with 10ml B27 supplements (Invitrogen, Life Technologies) plus glutamine (0.5mM), pyruvate, uridine and antibiotic-antimycotic, as previously described [45]. Staurosporine (4nM-8nM) dilutions were made fresh in DM and replaced every 2-3 days. Differentiation was completed on day 12 [45].

To measure mitochondrial movement, PD_Orig_ and PD_CLB_ cybrid neuronal cells were incubated with 50nM MitoTracker CMXRos (MTRed; Invitrogen, Life Technologies) for 10 min at 37°C. Time-lapse recordings were made using an Olympus IX70 microscope equipped with epifluorescence and Nomarski optics, a Lambda 10-2 filter wheel, a Photometrics CoolSnap HQ progressive scan CCD camera and a heater/controller to maintain cybrid cells at 37°C during image collection (World Precision Instruments, Inc). Image stacks and velocity measurements were collected using MetaMorph Imaging System (Molecular Devices). For standard recordings, images were collected every 3 seconds for 2 min. Mitochondrial movement was measured in PD61_Orig_ (n=4 cultures, 73 processes), PD61_CLB_ (n=4 cultures, 94 processes), PD67_Orig_ (n=4 cultures, 98 processes), PD67_CLB_ (n=5 cultures, 108 processes), PD63_Orig_ (n=8 cultures, 167 processes) and PD63_CLB_ (n=4 cultures, 80 processes). Unlike other studies, the velocity of all mitochondria in each cell process was tracked individually whether they moved or not. The average velocity for individual mitochondria was calculated using intervals where movement occurred. Neutral density filters are used to reduce illumination from the mercury lamp and minimize phototoxicity.

## Abbreviations

alpha-synuclein: α-synuclein; cDNA: Complimentary DNA; CLB: Cybrid lewy body, Cybrid lewy bodies; CNTL: Control; DM: Differentiation media; DMEM: Dulbecco’s modified eagle medium; DNA: Deoxyribonucleic acid; ECAR: Extracellular acidification rates; EM: Electron microscopy; ETC: Electron transport chain; FCCP: Carbonyl cyanide 4-(trifluoromethoxy)phenylhydrazone; gDNA: Genomic DNA; GM: Growth media; LB: Lewy body, Lewy bodies; LCM: Laser capture microscopy; mtDNA: Mitochondrial DNA; NRF1: Nuclear respiratory factor 1; OCR: Oxygen consumption rates; PD: Parkinson’s disease; PGC-1α: Peroxisome proliferator-activated receptor gamma coactivator 1-alpha; RNA: Ribonucleic acid; RT-qPCR: Quantitative real-time polymerase chain reaction; TFAM: Mitochondrial transcription factor A; TFB1M: Mitochondrial transcription factor B1.

## Competing interests

All authors declared that they have no competing interest.

## Authors’ contributions

ENC-F carried out experiments, performed statistical analysis and assisted in writing of the manuscript. MKB developed and carried out the cloning protocol and differentiated cells for axonal transport experiments. JPB designed primers and probes for RT-qPCR experiments and assisted in design and interpretation of the RT-qPCR experiments. KEB provided and maintained stocks of cybrid cell lines and carried out cell viability experiments. PAT carried out experiments, supervised and conceived of the presented research. All authors read and approved the final manuscript.

## Supplementary Material

Additional file 1 **Patient disease characteristics.** Demographics and disease characteristics for PD patients and controls used in this study Click here for file

Additional file 2 **CLB size and expression frequencies.** Congo red positive CLB size means, standard deviations, minimum and maximum sizes are shown, as well as expression frequency mean and standard deviations, described as a percent of total cells. There was no significant difference between the size or frequency means for any of the PD_Orig_ and PD_CLB_-selected pairs. Maximum size was also not significantly different between cell lines. Click here for file

Additional file 3 **CLB stained with antibodies for αlpha-synuclein and poly-ubiquitin.** In short, cells were plated in dishes and grown for 48-72 hours before being fixed and permeabilized using citrate antigen retrieval buffer. Dishes were blocked with 1%BSA/1%Triton blocking buffer and incubated in primary antibodies overnight at 4°. Dishes were then stained with fluorophore conjugated secondary antibodies (Life Technologies) and mounted using Vectashield mounting medium with DAPI (Vector Labs). Antibodies used: αlpha-synuclein (1:400, Millipore AB5038); poly-ubiquitin (1:200, Enzo BML-PW8805). Scale bar: 5μm. Click here for file

Additional file 4 **Extracellular CLB.** (A-B) Live PD cybrid cells showing extracellular CLB stained with Congo red. (C) Fixed PD cybrid cells stained with αlpha-synuclein (green) and poly-ubiquitin (red) to mark CLB. Nuclei shown are in blue. Scale bar: 10μm (A, B), 5μm (C); arrows: extracellular CLB. Click here for file

Additional file 5 **Neuronal viability after differentiation.** To determine neuronal viability for each cell line, 10 images were taken from 2 dishes of each differentiated PD cybrid pair with differentiated SH-SY5Y as a control. (A) Representative images for each cell line. Cells were counted in each image and calculated as cells per square centimeter and then normalized to cells per square millimeter originally plated in each dish. The normalized means from 2 dishes were combined and graphed (B). The mean per dish was substantially lower in PD63_CLB_ than PD63_Orig_. Scale bar: 10μm Click here for file

Additional file 6 **Gene expression of αlpha-synuclein.** αlpha-synuclein expression was measured using qRT-PCR. There was no difference in expression between PD_Orig_ and PD_CLB_ lines for any of the three pairs (Student’s *t*-test, n=3, p<0.05). Click here for file

Additional file 7 **Primer and probe sequences for qRT-PCR.** Primers and probes (Operon) were designed using Beacon Designer (Premier Biosoft). Click here for file

## References

[B1] Braak H Del TK Bratzke H Hamm-Clement J Sandmann-Keil D Rub U Staging of the intracerebral inclusion body pathology associated with idiopathic Parkinson’s disease (preclinical and clinical stages) J Neurol 2002 249 3 III/1 III/5 10.1007/s00415-002-1301-4 12528692

[B2] Burbulla LF Schelling C Kato H Rapaport D Woitalla D Schiesling C Schulte C Sharma M Illig T Bauer P Dissecting the role of the mitochondrial chaperone mortalin in Parkinson’s disease: functional impact of disease-related variants on mitochondrial homeostasis Hum Mol Genet 2010 19 4437 4452 10.1093/hmg/ddq370 20817635 PMC3298849

[B3] Trimmer PA Bennett JP Jr The cybrid model of sporadic Parkinson’s disease Exp Neurol 2009 218 320 325 10.1016/j.expneurol.2009.03.016 19328199 PMC2735256

[B4] Esteves AR Domingues AF Ferreira IL Januario C Swerdlow RH Oliveira CR Cardoso SM Mitochondrial function in Parkinson’s disease cybrids containing an nt2 neuron-like nuclear background Mitochondrion 2008 8 219 228 10.1016/j.mito.2008.03.004 18495557

[B5] Exner N Lutz AK Haass C Winklhofer KF Mitochondrial dysfunction in Parkinson’s disease: molecular mechanisms and pathophysiological consequences EMBO J 2012 31 3038 3062 10.1038/emboj.2012.170 22735187 PMC3400019

[B6] Esteves AR Lu J Rodova M Onyango I Lezi E Dubinsky R Lyons KE Pahwa R Burns JM Cardoso SM Swerdlow RH Mitochondrial respiration and respiration-associated proteins in cell lines created through Parkinson’s subject mitochondrial transfer J Neurochem 2010 113 674 682 10.1111/j.1471-4159.2010.06631.x 20132468

[B7] Keeney PM Dunham LD Quigley CK Morton SL Bergquist KE Bennett JP Jr Cybrid models of Parkinson’s disease show variable mitochondrial biogenesis and genotype-respiration relationships Exp Neurol 2009 220 374 382 10.1016/j.expneurol.2009.09.025 19815014 PMC2783275

[B8] Trimmer PA Borland MK Keeney PM Bennett JP Jr Parker WD Jr Parkinson’s disease transgenic mitochondrial cybrids generate Lewy inclusion bodies J Neurochem 2004 88 800 812 10.1046/j.1471-4159.2003.02168.x 14756800

[B9] Leverenz JB Umar I Wang Q Montine TJ McMillan PJ Tsuang DW Jin J Pan C Shin J Zhu D Zhang J Proteomic identification of novel proteins in cortical lewy bodies Brain Pathol 2007 17 139 145 10.1111/j.1750-3639.2007.00048.x 17388944 PMC8095629

[B10] Xia Q Liao L Cheng D Duong DM Gearing M Lah JJ Levey AI Peng J Proteomic identification of novel proteins associated with Lewy bodies Front Biosci 2008 13 3850 3856 18508479 10.2741/2973 PMC2663966

[B11] Wakabayashi K Tanji K Mori F Takahashi H The Lewy body in Parkinson’s disease: molecules implicated in the formation and degradation of alpha-synuclein aggregates Neuropathology 2007 27 494 506 10.1111/j.1440-1789.2007.00803.x 18018486

[B12] Galvin JE Lee VM Schmidt ML Tu PH Iwatsubo T Trojanowski JQ Pathobiology of the Lewy body Adv Neurol 1999 80 313 324 10410736

[B13] Shults CW Lewy bodies Proc Natl Acad Sci USA 2006 103 1661 1668 10.1073/pnas.0509567103 16449387 PMC1413649

[B14] Lu L Neff F Alvarez-Fischer D Henze C Xie Y Oertel WH Schlegel J Hartmann A Gene expression profiling of Lewy body-bearing neurons in Parkinson’s disease Exp Neurol 2005 195 27 39 10.1016/j.expneurol.2005.04.011 15944136

[B15] Conway KA Harper JD Lansbury PT Jr Fibrils formed in vitro from alpha-synuclein and two mutant forms linked to Parkinson’s disease are typical amyloid Biochemistry 2000 39 2552 2563 10.1021/bi991447r 10704204

[B16] Sanchez I Mahlke C Yuan J Pivotal role of oligomerization in expanded polyglutamine neurodegenerative disorders Nature 2003 421 373 379 10.1038/nature01301 12540902

[B17] Bertrand SJ Aksenova MV Aksenov MY Mactutus CF Booze RM Endogenous amyloidogenesis in long-term rat hippocampal cell cultures BMC Neurosci 2011 12 38 10.1186/1471-2202-12-38 21569253 PMC3112111

[B18] Dickson DW Parkinson’s disease and parkinsonism: neuropathology Cold Spring Harb Perspect Med 2012 2 a009258 22908195 10.1101/cshperspect.a009258 PMC3405828

[B19] Olanow CW Perl DP DeMartino GN McNaught KS Lewy-body formation is an aggresome-related process: a hypothesis Lancet Neurol 2004 3 496 503 10.1016/S1474-4422(04)00827-0 15261611

[B20] Lees AJ Hardy J Revesz T Parkinson’s disease Lancet 2009 373 2055 2066 10.1016/S0140-6736(09)60492-X 19524782

[B21] Greffard S Verny M Bonnet AM Seilhean D Hauw JJ Duyckaerts C A stable proportion of Lewy body bearing neurons in the substantia nigra suggests a model in which the Lewy body causes neuronal death Neurobiol Aging 2010 31 99 103 10.1016/j.neurobiolaging.2008.03.015 18457903

[B22] Rujano MA Bosveld F Salomons FA Dijk F van Waarde MA van der Want JJ de Vos RA Brunt ER Sibon OC Kampinga HH Polarised asymmetric inheritance of accumulated protein damage in higher eukaryotes PLoS Biol 2006 4 e417 10.1371/journal.pbio.0040417 17147470 PMC1750924

[B23] Fuentealba LC Eivers E Geissert D Taelman V De Robertis EM Asymmetric mitosis: Unequal segregation of proteins destined for degradation Proc Natl Acad Sci USA 2008 105 7732 7737 10.1073/pnas.0803027105 18511557 PMC2402384

[B24] Alafuzoff I Ince PG Arzberger T Al-Sarraj S Bell J Bodi I Bogdanovic N Bugiani O Ferrer I Gelpi E Staging/typing of Lewy body related alpha-synuclein pathology: a study of the BrainNet Europe Consortium Acta Neuropathol 2009 117 635 652 10.1007/s00401-009-0523-2 19330340

[B25] Doehner J Genoud C Imhof C Krstic D Knuesel I Extrusion of misfolded and aggregated proteins - a protective strategy of aging neurons? Eur J Neurosci 2012 35 1938 1950 10.1111/j.1460-9568.2012.08154.x 22708604

[B26] Campello S Scorrano L Mitochondrial shape changes: orchestrating cell pathophysiology EMBO Rep 2010 11 678 684 10.1038/embor.2010.115 20725092 PMC2933866

[B27] Trimmer PA Swerdlow RH Parks JK Keeney P Bennett JP Jr Miller SW Davis RE Parker WD Jr Abnormal mitochondrial morphology in sporadic Parkinson’s and Alzheimer’s disease cybrid cell lines Exp Neurol 2000 162 37 50 10.1006/exnr.2000.7333 10716887

[B28] Knott AB Perkins G Schwarzenbacher R Bossy-Wetzel E Mitochondrial fragmentation in neurodegeneration Nat Rev Neurosci 2008 9 505 518 18568013 10.1038/nrn2417 PMC2711514

[B29] Gomes LC Di Benedetto G Scorrano L During autophagy mitochondria elongate, are spared from degradation and sustain cell viability Nat Cell Biol 2011 13 589 598 10.1038/ncb2220 21478857 PMC3088644

[B30] Twig G Hyde B Shirihai OS Mitochondrial fusion, fission and autophagy as a quality control axis: the bioenergetic view Biochim Biophys Acta 2008 1777 1092 1097 10.1016/j.bbabio.2008.05.001 18519024 PMC3809017

[B31] Palmer CS Osellame LD Stojanovski D Ryan MT The regulation of mitochondrial morphology: intricate mechanisms and dynamic machinery Cell Signal 2011 23 1534 1545 10.1016/j.cellsig.2011.05.021 21683788

[B32] Zick M Rabl R Reichert AS Cristae formation-linking ultrastructure and function of mitochondria Biochim Biophys Acta 2009 1793 5 19 10.1016/j.bbamcr.2008.06.013 18620004

[B33] Alberts B Molecular biology of the cell 1983 New York: Garland Pub

[B34] Safiulina D Veksler V Zharkovsky A Kaasik A Loss of mitochondrial membrane potential is associated with increase in mitochondrial volume: physiological role in neurones J Cell Physiol 2006 206 347 353 10.1002/jcp.20476 16110491

[B35] Ferrick DA Neilson A Beeson C Advances in measuring cellular bioenergetics using extracellular flux Drug Discov Today 2008 13 268 274 10.1016/j.drudis.2007.12.008 18342804

[B36] Dranka BP Benavides GA Diers AR Giordano S Zelickson BR Reily C Zou L Chatham JC Hill BG Zhang J Assessing bioenergetic function in response to oxidative stress by metabolic profiling Free Radic Biol Med 2011 51 1621 1635 10.1016/j.freeradbiomed.2011.08.005 21872656 PMC3548422

[B37] Brand MD Nicholls DG Assessing mitochondrial dysfunction in cells Biochem J 2011 435 297 312 10.1042/BJ20110162 21726199 PMC3076726

[B38] Dranka BP Zielonka J Kanthasamy AG Kalyanaraman B Alterations in bioenergetic function induced by Parkinson’s disease mimetic compounds: lack of correlation with superoxide generation J Neurochem 2012 122 941 951 10.1111/j.1471-4159.2012.07836.x 22708893 PMC3423581

[B39] Chu Y Morfini GA Langhamer LB He Y Brady ST Kordower JH Alterations in axonal transport motor proteins in sporadic and experimental Parkinson’s disease Brain 2012 135 2058 2073 10.1093/brain/aws133 22719003 PMC4571141

[B40] Sekine S Miura M Chihara T Organelles in developing neurons: essential regulators of neuronal morphogenesis and function Int J Dev Biol 2009 53 19 27 10.1387/ijdb.082618ss 19123123

[B41] Cai Q Davis ML Sheng ZH Regulation of axonal mitochondrial transport and its impact on synaptic transmission Neurosci Res 2011 70 9 15 10.1016/j.neures.2011.02.005 21352858 PMC3086944

[B42] Kanazawa T Uchihara T Takahashi A Nakamura A Orimo S Mizusawa H Three-layered structure shared between Lewy bodies and lewy neurites-three-dimensional reconstruction of triple-labeled sections Brain Pathol 2008 18 415 422 10.1111/j.1750-3639.2008.00140.x 18394008 PMC8095600

[B43] Saxton WM Hollenbeck PJ The axonal transport of mitochondria J Cell Sci 2012 125 2095 2104 10.1242/jcs.053850 22619228 PMC3656622

[B44] Trimmer PA Schwartz KM Borland MK De Taboada L Streeter J Oron U Reduced axonal transport in Parkinson’s disease cybrid neurites is restored by light therapy Mol Neurodegener 2009 4 26 10.1186/1750-1326-4-26 19534794 PMC2711937

[B45] Borland MK Trimmer PA Rubinstein JD Keeney PM Mohanakumar K Liu L Bennett JP Jr Chronic, low-dose rotenone reproduces Lewy neurites found in early stages of Parkinson’s disease, reduces mitochondrial movement and slowly kills differentiated SH-SY5Y neural cells Mol Neurodegener 2008 3 21 10.1186/1750-1326-3-21 19114014 PMC2631511

[B46] Ashley N Harris D Poulton J Detection of mitochondrial DNA depletion in living human cells using PicoGreen staining Exp Cell Res 2005 303 432 446 10.1016/j.yexcr.2004.10.013 15652355

[B47] Bogenhagen DF Rousseau D Burke S The layered structure of human mitochondrial DNA nucleoids J Biol Chem 2008 283 3665 3675 18063578 10.1074/jbc.M708444200

[B48] He J Cooper HM Reyes A Di Re M Sembongi H Litwin TR Gao J Neuman KC Fearnley IM Spinazzola A Mitochondrial nucleoid interacting proteins support mitochondrial protein synthesis Nucleic Acids Res 2012 40 6109 6121 10.1093/nar/gks266 22453275 PMC3401451

[B49] Keeney PM Xie J Capaldi RA Bennett JP Jr Parkinson’s disease brain mitochondrial complex I has oxidatively damaged subunits and is functionally impaired and misassembled J Neurosci 2006 26 5256 5264 10.1523/JNEUROSCI.0984-06.2006 16687518 PMC6674236

[B50] Borland MK Mohanakumar KP Rubinstein JD Keeney PM Xie J Capaldi R Dunham LD Trimmer PA Bennett JP Jr Relationships among molecular genetic and respiratory properties of Parkinson’s disease cybrid cells show similarities to Parkinson’s brain tissues Biochim Biophys Acta 2008 1792 68 74 18973805 10.1016/j.bbadis.2008.09.014 PMC2655102

[B51] Moran M Moreno-Lastres D Marin-Buera L Arenas J Martin MA Ugalde C Mitochondrial respiratory chain dysfunction: Implications in neurodegeneration Free Radic Biol Med 2012 53 595 609 10.1016/j.freeradbiomed.2012.05.009 22595027

[B52] Bereiter-Hahn JaB M Distribution and dynamics of mitochondrial nucleoids in animal cells in culture Exp Biol Online 1997 1 1 17

[B53] Wareski P Vaarmann A Choubey V Safiulina D Liiv J Kuum M Kaasik A PGC-1{alpha} and PGC-1{beta} regulate mitochondrial density in neurons J Biol Chem 2009 284 21379 21385 10.1074/jbc.M109.018911 19542216 PMC2755862

[B54] Zheng B Liao Z Locascio JJ Lesniak KA Roderick SS Watt ML Eklund AC Zhang-James Y Kim PD Hauser MA PGC-1alpha, a potential therapeutic target for early intervention in Parkinson’s disease Sci Transl Med 2010 2 52 73 10.1126/scitranslmed.3001059 PMC3129986 20926834

[B55] Hock MB Kralli A Transcriptional control of mitochondrial biogenesis and function Annu Rev Physiol 2009 71 177 203 10.1146/annurev.physiol.010908.163119 19575678

[B56] Mudo G Makela J Di Liberto V Tselykh TV Olivieri M Piepponen P Eriksson O Malkia A Bonomo A Kairisalo M Transgenic expression and activation of PGC-1alpha protect dopaminergic neurons in the MPTP mouse model of Parkinson’s disease Cell Mol Life Sci: CMLS 2012 69 1153 1165 10.1007/s00018-011-0850-z PMC11114858 21984601

[B57] Srivastava S Diaz F Iommarini L Aure K Lombes A Moraes CT PGC-1alpha/beta induced expression partially compensates for respiratory chain defects in cells from patients with mitochondrial disorders Hum Mol Genet 2009 18 1805 1812 10.1093/hmg/ddp093 19297390 PMC2722224

[B58] Bastin J Aubey F Rotig A Munnich A Djouadi F Activation of peroxisome proliferator-activated receptor pathway stimulates the mitochondrial respiratory chain and can correct deficiencies in patients’ cells lacking its components J Clin Endocrinol Metab 2008 93 1433 1441 10.1210/jc.2007-1701 18211970

[B59] Wenz T Diaz F Spiegelman BM Moraes CT Activation of the PPAR/PGC-1alpha pathway prevents a bioenergetic deficit and effectively improves a mitochondrial myopathy phenotype Cell Metab 2008 8 249 256 10.1016/j.cmet.2008.07.006 18762025 PMC2613643

[B60] Scarpulla RC Nuclear control of respiratory chain expression by nuclear respiratory factors and PGC-1-related coactivator Ann N Y Acad Sci 2008 1147 321 334 10.1196/annals.1427.006 19076454 PMC2853241

[B61] Gaspari M Larsson NG Gustafsson CM The transcription machinery in mammalian mitochondria Biochim Biophys Acta 2004 1659 148 152 10.1016/j.bbabio.2004.10.003 15576046

[B62] Diaz F Moraes CT Mitochondrial biogenesis and turnover Cell Calcium 2008 44 24 35 10.1016/j.ceca.2007.12.004 18395251 PMC3175594

[B63] Wenz T Mitochondria and PGC-1alpha in Aging and Age-Associated Diseases J Aging Res 2011 2011 810619 21629705 10.4061/2011/810619 PMC3100651

[B64] Jellinger KA Formation and development of Lewy pathology: a critical update J Neurol 2009 256 Suppl 3 270 279 19711116 10.1007/s00415-009-5243-y

[B65] Harding AJ Broe GA Halliday GM Visual hallucinations in Lewy body disease relate to Lewy bodies in the temporal lobe Brain 2002 125 391 403 10.1093/brain/awf033 11844739

[B66] Harding AJ Stimson E Henderson JM Halliday GM Clinical correlates of selective pathology in the amygdala of patients with Parkinson’s disease Brain 2002 125 2431 2445 10.1093/brain/awf251 12390970

[B67] Harrower TP Michell AW Barker RA Lewy bodies in Parkinson’s disease: protectors or perpetrators? Exp Neurol 2005 195 1 6 10.1016/j.expneurol.2005.06.002 16023637

[B68] Harrower T Barker RA Cell therapies for neurological disease–from bench to clinic to bench Expert Opin Biol Ther 2005 5 289 291 10.1517/14712598.5.3.289 15833067

[B69] de la Fuente-Fernandez R Schulzer M Mak E Kishore A Calne DB The role of the Lewy body in idiopathic Parkinsonism Parkinsonism Relat Disord 1998 4 73 77 10.1016/S1353-8020(98)00016-9 18591092

[B70] Hindle JV Ageing, neurodegeneration and Parkinson’s disease Age Ageing 2010 39 156 161 10.1093/ageing/afp223 20051606

[B71] Au WL Calne DB A reassessment of the Lewy body Acta Neurol Taiwan 2005 14 40 47 16008161

[B72] Kramer ML Schulz-Schaeffer WJ Presynaptic alpha-synuclein aggregates, not Lewy bodies, cause neurodegeneration in dementia with Lewy bodies J Neurosci: Offic J Soc Neurosci 2007 27 1405 1410 10.1523/JNEUROSCI.4564-06.2007 PMC6673583 17287515

[B73] Zhou J Broe M Huang Y Anderson JP Gai WP Milward EA Porritt M Howells D Hughes AJ Wang X Halliday GM Changes in the solubility and phosphorylation of alpha-synuclein over the course of Parkinson’s disease Acta Neuropathol 2011 121 695 704 10.1007/s00401-011-0815-1 21400129

[B74] Lin CJ Lee CC Shih YL Lin CH Wang SH Chen TH Shih CM Inhibition of mitochondria- and endoplasmic reticulum stress-mediated autophagy augments temozolomide-induced apoptosis in glioma cells PLoS One 2012 7 e38706 10.1371/journal.pone.0038706 22745676 PMC3382156

[B75] Devine MJ Gwinn K Singleton A Hardy J Parkinson’s disease and alpha-synuclein expression Mov Disord 2011 26 2160 2168 10.1002/mds.23948 21887711 PMC4669565

[B76] Ferrer I Neuropathology and neurochemistry of nonmotor symptoms in Parkinson’s disease Parkinsons Dis 2011 2011 708404 21403906 10.4061/2011/708404 PMC3043318

[B77] Siddiqui A Chinta SJ Mallajosyula JK Rajagopolan S Hanson I Rane A Melov S Andersen JK Selective binding of nuclear alpha-synuclein to the PGC1alpha promoter under conditions of oxidative stress may contribute to losses in mitochondrial function: Implications for Parkinson’s disease Free Radic Biol Med 2012 53 993 1003 10.1016/j.freeradbiomed.2012.05.024 22705949 PMC3418424

[B78] Swerdlow RH Parks JK Miller SW Tuttle JB Trimmer PA Sheehan JP Bennett JP Jr Davis RE Parker WD Jr Origin and functional consequences of the complex I defect in Parkinson’s disease Ann Neurol 1996 40 663 671 10.1002/ana.410400417 8871587

[B79] Miller SW Trimmer PA Parker WD Jr Davis RE Creation and characterization of mitochondrial DNA-depleted cell lines with "neuronal-like" properties J Neurochem 1996 67 1897 1907 8863494 10.1046/j.1471-4159.1996.67051897.x

