# Supplementary material for: Mitochondrial quality, dynamics and functional capacity in Parkinson’s disease cybrid cell lines selected for Lewy body expression
Source: Mol Neurodegener. 2013 Jan 26;8:6. doi: 10.1186/1750-1326-8-6 (PMC3577453; doi:10.1186/1750-1326-8-6)
Supplement: Additional file 1 — Patient disease characteristics. Demographics and disease characteristics for PD patients and controls used in this study [file 1750-1326-8-6-S1.ppt]

## Slide 1
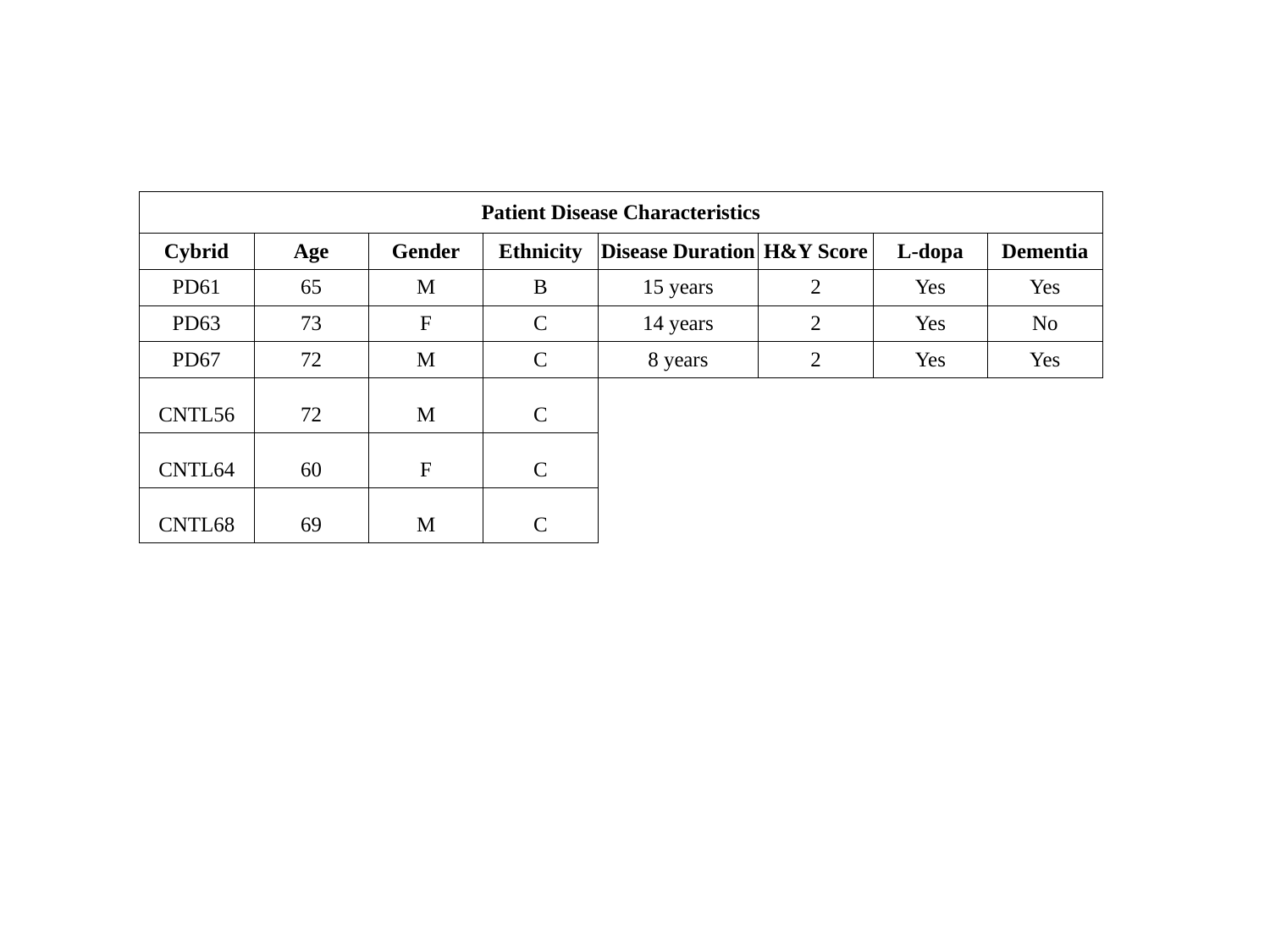

| Patient Disease Characteristics | | | | | | | |
| --- | --- | --- | --- | --- | --- | --- | --- |
| Cybrid | Age | Gender | Ethnicity | Disease Duration | H&Y Score | L-dopa | Dementia |
| PD61 | 65 | M | B | 15 years | 2 | Yes | Yes |
| PD63 | 73 | F | C | 14 years | 2 | Yes | No |
| PD67 | 72 | M | C | 8 years | 2 | Yes | Yes |
| CNTL56 | 72 | M | C | | | | |
| CNTL64 | 60 | F | C | | | | |
| CNTL68 | 69 | M | C | | | | |
