# Supplementary material for: Mitochondrial quality, dynamics and functional capacity in Parkinson’s disease cybrid cell lines selected for Lewy body expression
Source: Mol Neurodegener. 2013 Jan 26;8:6. doi: 10.1186/1750-1326-8-6 (PMC3577453; doi:10.1186/1750-1326-8-6)
Supplement: Additional file 2 — CLB size and expression frequencies. Congo red positive CLB size means, standard deviations, minimum and maximum sizes are shown, as well as expression frequency mean and standard deviations, described as a percent of total cells. There was no significant difference between the size or frequency means for any of the PDOrig and PDCLB-selected pairs. Maximum size was also not significantly different between cell lines. [file 1750-1326-8-6-S2.ppt]

## Slide 1
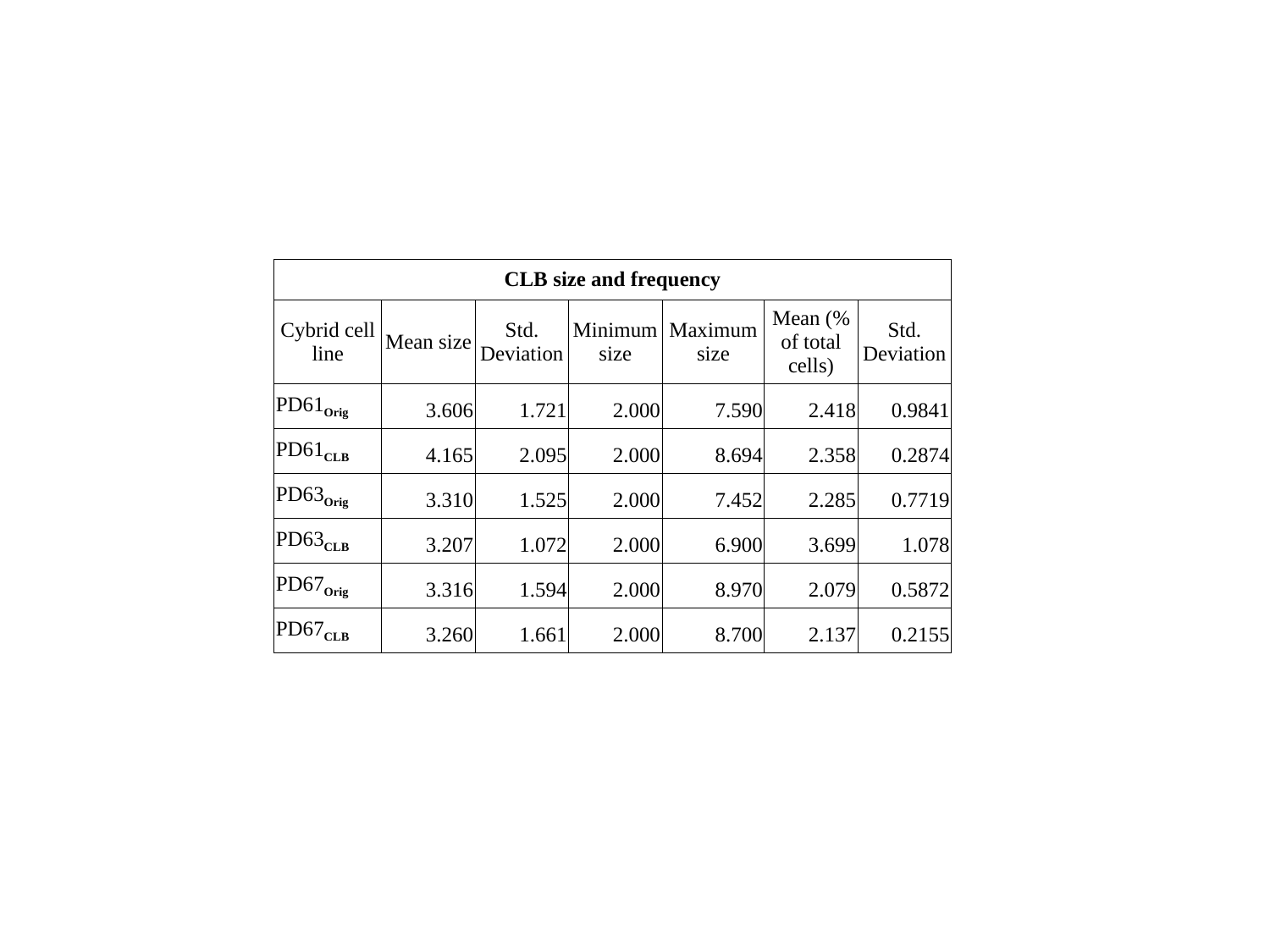

| CLB size and frequency | | | | | | |
| --- | --- | --- | --- | --- | --- | --- |
| Cybrid cell line | Mean size | Std. Deviation | Minimum size | Maximum size | Mean (% of total cells) | Std. Deviation |
| PD61Orig | 3.606 | 1.721 | 2.000 | 7.590 | 2.418 | 0.9841 |
| PD61CLB | 4.165 | 2.095 | 2.000 | 8.694 | 2.358 | 0.2874 |
| PD63Orig | 3.310 | 1.525 | 2.000 | 7.452 | 2.285 | 0.7719 |
| PD63CLB | 3.207 | 1.072 | 2.000 | 6.900 | 3.699 | 1.078 |
| PD67Orig | 3.316 | 1.594 | 2.000 | 8.970 | 2.079 | 0.5872 |
| PD67CLB | 3.260 | 1.661 | 2.000 | 8.700 | 2.137 | 0.2155 |
