# Supplementary material for: Mitochondrial quality, dynamics and functional capacity in Parkinson’s disease cybrid cell lines selected for Lewy body expression
Source: Mol Neurodegener. 2013 Jan 26;8:6. doi: 10.1186/1750-1326-8-6 (PMC3577453; doi:10.1186/1750-1326-8-6)
Supplement: Additional file 3 — CLB stained with antibodies for αlpha-synuclein and poly-ubiquitin. In short, cells were plated in dishes and grown for 48-72 hours before being fixed and permeabilized using citrate antigen retrieval buffer. Dishes were blocked with 1%BSA/1%Triton blocking buffer and incubated in primary antibodies overnight at 4°. Dishes were then stained with fluorophore conjugated secondary antibodies (Life Technologies) and mounted using Vectashield mounting medium with DAPI (Vector Labs). Antibodies used: αlpha-synuclein (1:400, Millipore AB5038); poly-ubiquitin (1:200, Enzo BML-PW8805). Scale bar: 5μm. [file 1750-1326-8-6-S3.ppt]

## Slide 1
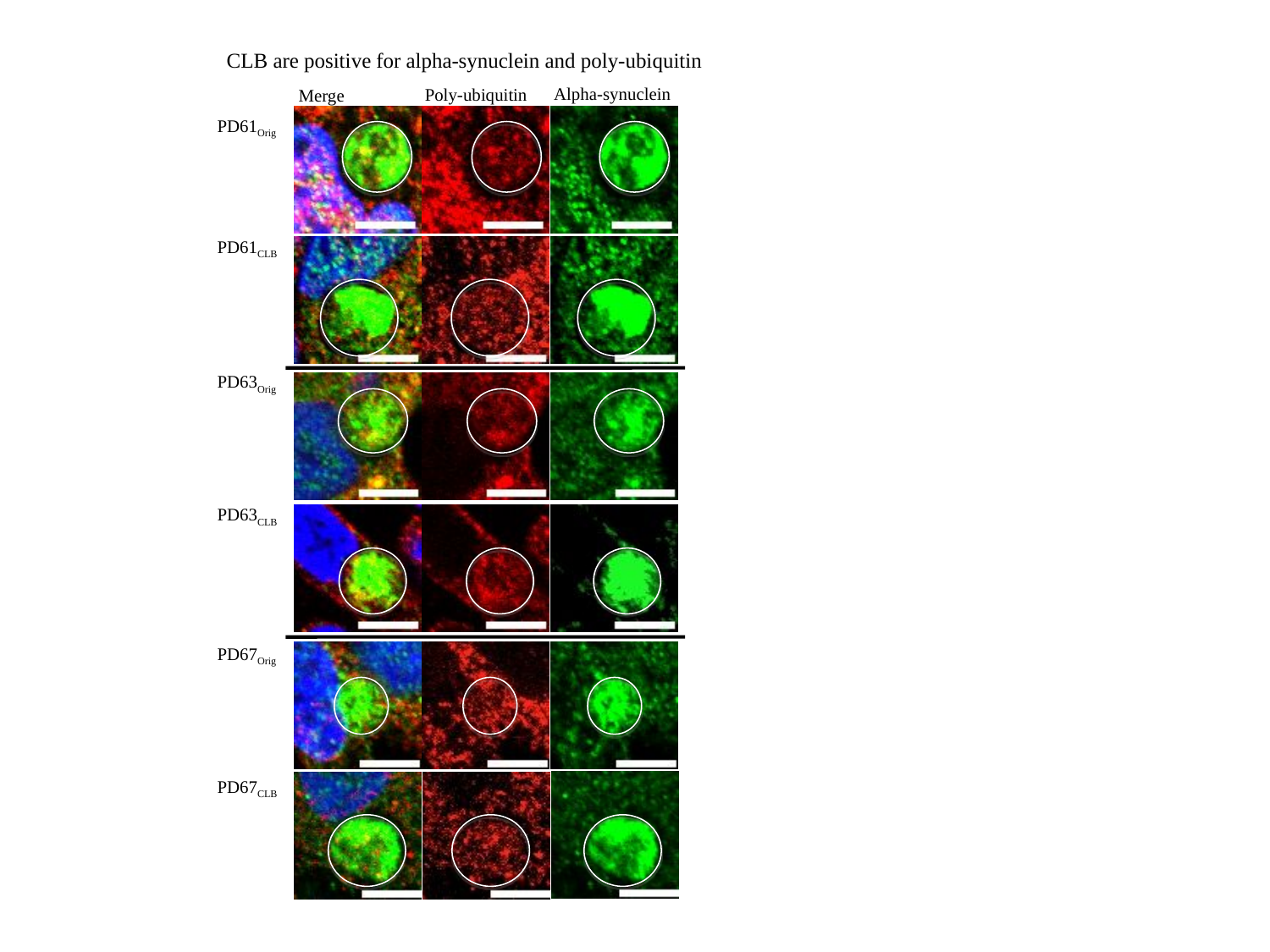

CLB are positive for alpha-synuclein and poly-ubiquitin
Alpha-synuclein
Poly-ubiquitin
Merge
PD61Orig
PD61CLB
PD63Orig
PD63CLB
PD67Orig
PD67CLB
