# Supplementary figures and images for: Mitochondrial quality, dynamics and functional capacity in Parkinson’s disease cybrid cell lines selected for Lewy body expression
Source: Mol Neurodegener. 2013 Jan 26;8:6. doi: 10.1186/1750-1326-8-6 (PMC3577453; doi:10.1186/1750-1326-8-6)

## Slide 1
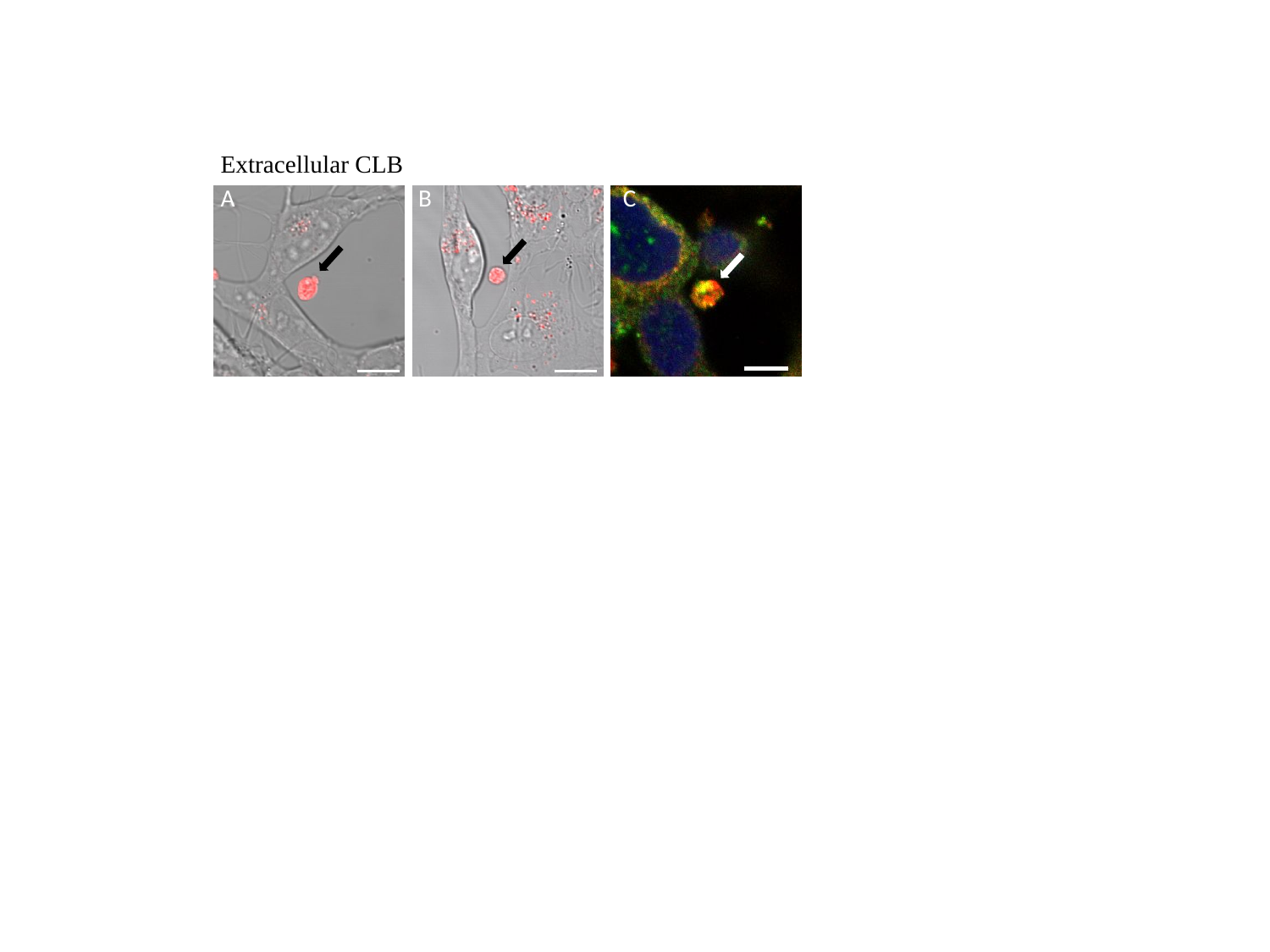

Extracellular CLB
A
B
C

Supplement: Additional file 4 — Extracellular CLB. (A-B) Live PD cybrid cells showing extracellular CLB stained with Congo red. (C) Fixed PD cybrid cells stained with αlpha-synuclein (green) and poly-ubiquitin (red) to mark CLB. Nuclei shown are in blue. Scale bar: 10μm (A, B), 5μm (C); arrows: extracellular CLB. [file 1750-1326-8-6-S4.ppt]
