# Supplementary material for: Mitochondrial quality, dynamics and functional capacity in Parkinson’s disease cybrid cell lines selected for Lewy body expression
Source: Mol Neurodegener. 2013 Jan 26;8:6. doi: 10.1186/1750-1326-8-6 (PMC3577453; doi:10.1186/1750-1326-8-6)
Supplement: Additional file 5 — Neuronal viability after differentiation. To determine neuronal viability for each cell line, 10 images were taken from 2 dishes of each differentiated PD cybrid pair with differentiated SH-SY5Y as a control. (A) Representative images for each cell line. Cells were counted in each image and calculated as cells per square centimeter and then normalized to cells per square millimeter originally plated in each dish. The normalized means from 2 dishes were combined and graphed (B). The mean per dish was substantially lower in PD63CLB than PD63Orig. Scale bar: 10μm [file 1750-1326-8-6-S5.ppt]

## Slide 1
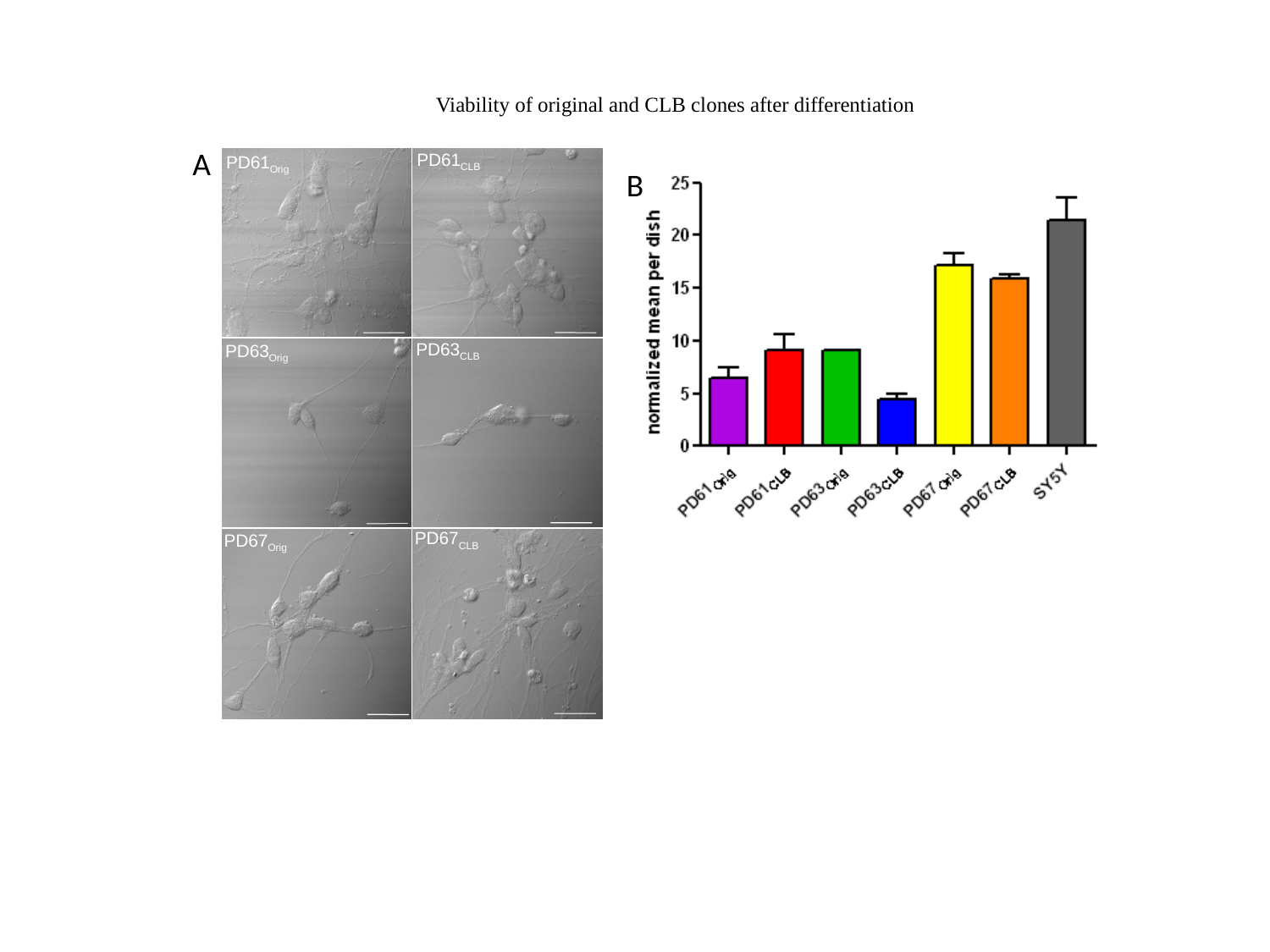

Viability of original and CLB clones after differentiation
A
PD61CLB
PD61Orig
B
PD63CLB
PD63Orig
PD67CLB
PD67Orig
