# Supplementary material for: Mitochondrial quality, dynamics and functional capacity in Parkinson’s disease cybrid cell lines selected for Lewy body expression
Source: Mol Neurodegener. 2013 Jan 26;8:6. doi: 10.1186/1750-1326-8-6 (PMC3577453; doi:10.1186/1750-1326-8-6)
Supplement: Additional file 6 — Gene expression of αlpha-synuclein. αlpha-synuclein expression was measured using qRT-PCR. There was no difference in expression between PDOrig and PDCLB lines for any of the three pairs (Student’s t-test, n=3, p<0.05). [file 1750-1326-8-6-S6.ppt]

## Slide 1
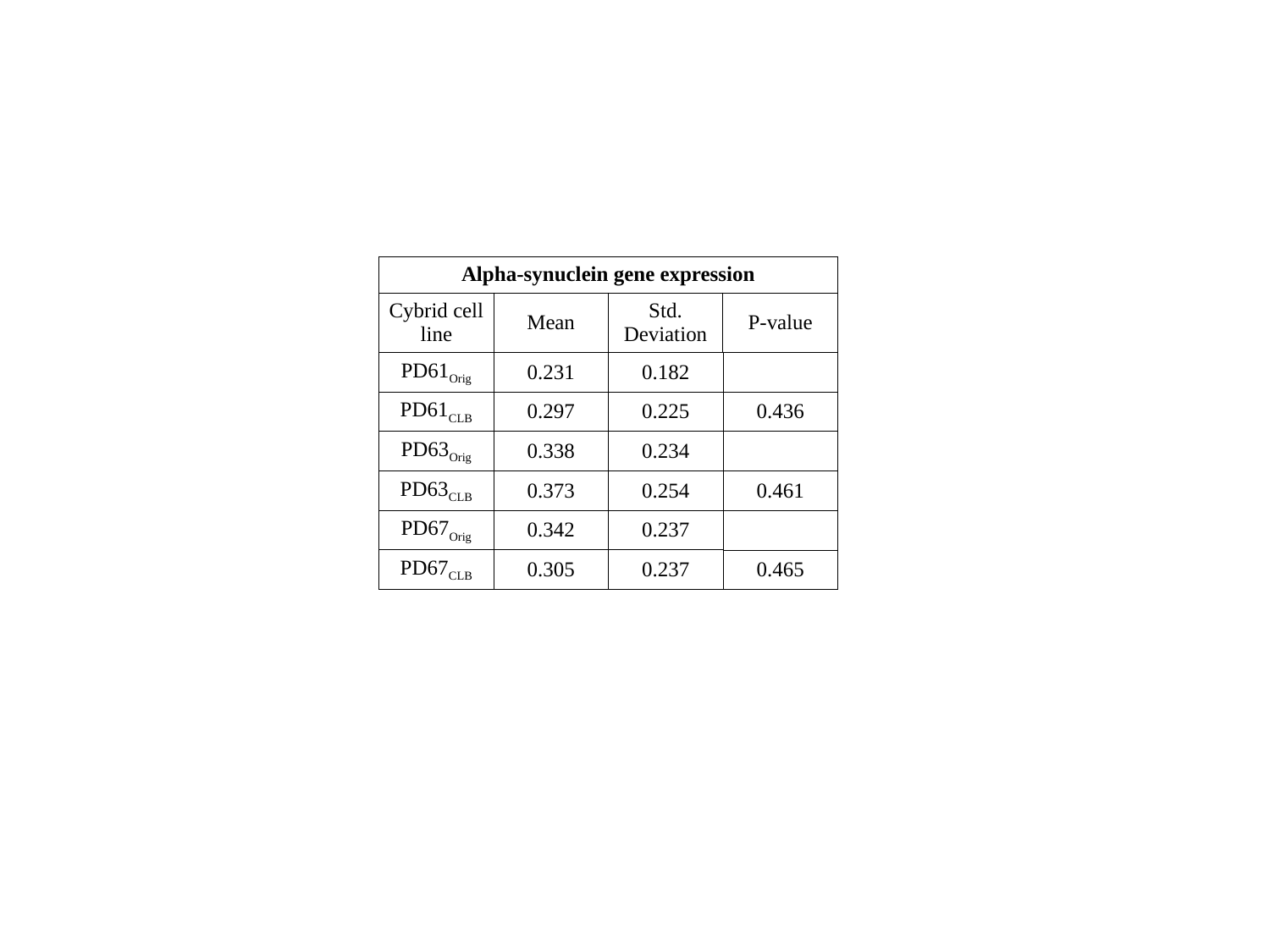

| Alpha-synuclein gene expression | | | |
| --- | --- | --- | --- |
| Cybrid cell line | Mean | Std. Deviation | P-value |
| PD61Orig | 0.231 | 0.182 | |
| PD61CLB | 0.297 | 0.225 | 0.436 |
| PD63Orig | 0.338 | 0.234 | |
| PD63CLB | 0.373 | 0.254 | 0.461 |
| PD67Orig | 0.342 | 0.237 | |
| PD67CLB | 0.305 | 0.237 | 0.465 |
