# Supplementary material for: Mitochondrial quality, dynamics and functional capacity in Parkinson’s disease cybrid cell lines selected for Lewy body expression
Source: Mol Neurodegener. 2013 Jan 26;8:6. doi: 10.1186/1750-1326-8-6 (PMC3577453; doi:10.1186/1750-1326-8-6)
Supplement: Additional file 7 — Primer and probe sequences for qRT-PCR. Primers and probes (Operon) were designed using Beacon Designer (Premier Biosoft). [file 1750-1326-8-6-S7.ppt]

## Slide 1
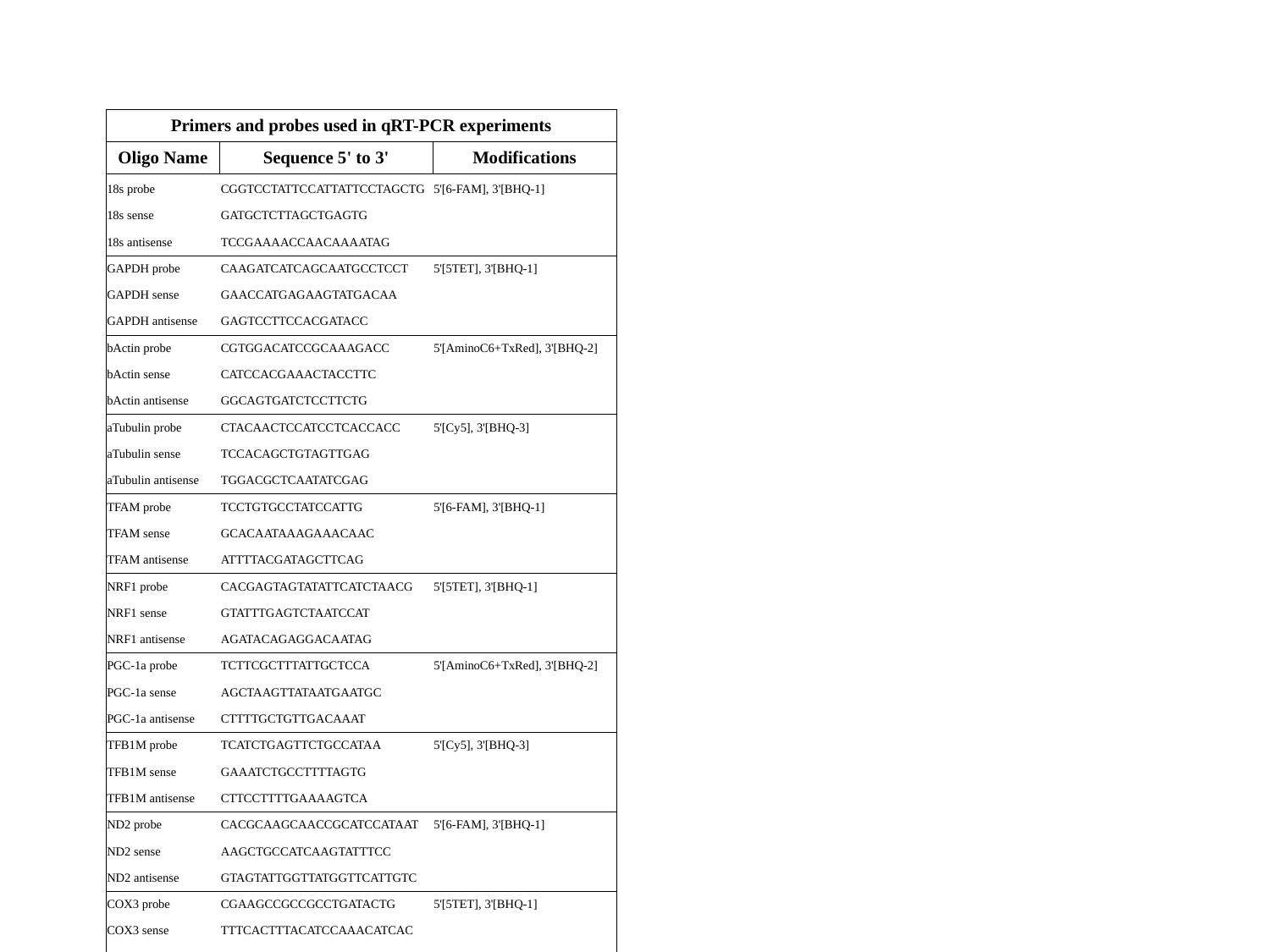

| Primers and probes used in qRT-PCR experiments | | |
| --- | --- | --- |
| Oligo Name | Sequence 5' to 3' | Modifications |
| 18s probe | CGGTCCTATTCCATTATTCCTAGCTG | 5'[6-FAM], 3'[BHQ-1] |
| 18s sense | GATGCTCTTAGCTGAGTG | |
| 18s antisense | TCCGAAAACCAACAAAATAG | |
| GAPDH probe | CAAGATCATCAGCAATGCCTCCT | 5'[5TET], 3'[BHQ-1] |
| GAPDH sense | GAACCATGAGAAGTATGACAA | |
| GAPDH antisense | GAGTCCTTCCACGATACC | |
| bActin probe | CGTGGACATCCGCAAAGACC | 5'[AminoC6+TxRed], 3'[BHQ-2] |
| bActin sense | CATCCACGAAACTACCTTC | |
| bActin antisense | GGCAGTGATCTCCTTCTG | |
| aTubulin probe | CTACAACTCCATCCTCACCACC | 5'[Cy5], 3'[BHQ-3] |
| aTubulin sense | TCCACAGCTGTAGTTGAG | |
| aTubulin antisense | TGGACGCTCAATATCGAG | |
| TFAM probe | TCCTGTGCCTATCCATTG | 5'[6-FAM], 3'[BHQ-1] |
| TFAM sense | GCACAATAAAGAAACAAC | |
| TFAM antisense | ATTTTACGATAGCTTCAG | |
| NRF1 probe | CACGAGTAGTATATTCATCTAACG | 5'[5TET], 3'[BHQ-1] |
| NRF1 sense | GTATTTGAGTCTAATCCAT | |
| NRF1 antisense | AGATACAGAGGACAATAG | |
| PGC-1a probe | TCTTCGCTTTATTGCTCCA | 5'[AminoC6+TxRed], 3'[BHQ-2] |
| PGC-1a sense | AGCTAAGTTATAATGAATGC | |
| PGC-1a antisense | CTTTTGCTGTTGACAAAT | |
| TFB1M probe | TCATCTGAGTTCTGCCATAA | 5'[Cy5], 3'[BHQ-3] |
| TFB1M sense | GAAATCTGCCTTTTAGTG | |
| TFB1M antisense | CTTCCTTTTGAAAAGTCA | |
| ND2 probe | CACGCAAGCAACCGCATCCATAAT | 5'[6-FAM], 3'[BHQ-1] |
| ND2 sense | AAGCTGCCATCAAGTATTTCC | |
| ND2 antisense | GTAGTATTGGTTATGGTTCATTGTC | |
| COX3 probe | CGAAGCCGCCGCCTGATACTG | 5'[5TET], 3'[BHQ-1] |
| COX3 sense | TTTCACTTTACATCCAAACATCAC | |
| COX3 antisense | CAATAGATGGAGACATACAGAAATAG | |
| ND4 probe | AGCCAGAACGCCTGAACGCAG | 5'[AminoC6+TxRed], 3'[BHQ-2] |
| ND4 sense | TGGCTATCATCACCCGATG | |
| ND4 antisense | TGAGTAGTAGAATGTTTAGTGAGC | |
| 12s probe | CGCCAGAACACTACGAGCCACAG | 5'[dQuasara670], 3'[BHQ-3] |
| 12s sense | CCTCAACAGTTAAATCAACAAAAC | |
| 12s antisense | CTGAGCAAGAGGTGGTGAG | |
